# The application of late amniocentesis: a retrospective study in a tertiary fetal medicine center in China

**DOI:** 10.1186/s12884-021-03723-7

**Published:** 2021-03-30

**Authors:** Yingting Li, Huanchen Yan, Jingsi Chen, Fei Chen, Wei Jian, Jiayan Wang, Xiaoqing Ye, Yufan Li, Nan Li, Philip C. N. Chiu, Min Chen

**Affiliations:** 1 Department of Obstetrics and Gynecology, Department of Fetal Medicine and Prenatal Diagnosis, Key Laboratory for Major Obstetric Diseases of Guangdong Province, The Third Affiliated Hospital of Guangzhou Medical University, 63 Duobao Road, Liwan District, Guangzhou, China; 2 Department of Obstetrics and Gynecology, the University of Hong Kong, Hong Kong, China; 3 Shenzhen Key Laboratory of Fertility Regulation, the University of Hong Kong-Shenzhen Hospital, Shenzhen, China

**Keywords:** Prenatal diagnosis, Chromosomal microarray analysis, Fetal abnormalities, Late amniocentesis, Whole-exome sequencing, Third trimester

## Abstract

**Background:**

To assess the indications and complications of late amniocentesis and the advanced genetic test results in a tertiary university fetal medical medicine unit.

**Methods:**

In this retrospective study, women that underwent amniocentesis at 24^+ 0^ to 39^+ 4^ weeks, between January 2014 and December 2019, were recruited. Indications, complications, genetic test results, and pregnancy outcomes were reported for each pregnancy and compared with those who underwent the traditional amniocentesis at 16^+ 0^ to 23^+ 6^ weeks (control group). Information was retrieved from patient medical records, checked by research staff, and analyzed.

**Results:**

Of the 1287 women (1321 fetuses) included in the late amniocentesis group, late detected sonographic abnormalities (85.5%) were the most common indication. The overall incidence of preterm birth and intrauterine demise after amniocentesis were 2.5 and 1.3%, respectively. Sixty-nine fetuses with aneuploidy (5.3%) and seventy-two fetuses with pathogenic copy number variations (5.5%) were identified by chromosomal microarray analysis. The maximal diagnostic yield (70%) was in the subgroup of fetuses with the abnormal diagnostic test results, followed by abnormal NIPT results (35.7%) and multiple abnormalities (23.8%). And 35.4% of the pregnancies were finally terminated.

**Conclusions:**

Due to the high detection rates of advanced genetic technologies and the safety of the invasive procedure (3.9% vs 4.0%), it is reasonable to recommend late amniocentesis as an effective and reliable method to detect late-onset fetal abnormalities. However, chromosomal microarray and whole-exome sequencing may result in uncertain results like variants of uncertain significance. Comprehensive genetic counseling is necessary.

**Supplementary Information:**

The online version contains supplementary material available at 10.1186/s12884-021-03723-7.

## Introduction

Amniocentesis is a procedure in which a small amount of amniotic fluid is withdrawn from the sac surrounding the fetus for testing in prenatal diagnosis. Since the late 1960s, amniocentesis became a widely accepted method of obtaining fetal genetic information [1]. It is conventionally performed between 16-24 gestational weeks, and it provides pregnant women and their families with an opportunity for early diagnosis of the undesirable findings and appropriate intervention for the pregnancies.

Nowadays, clinically available methods for analyzing fetal genetic information from amniotic fluid include traditional karyotyping, chromosomal microarray analysis (CMA), whole-genome sequencing (WGS), and whole-exome sequencing (WES). The detection ranges of them are different, with different detection rates and costs. For example, compared with traditional karyotyping, CMA provides more fetal genomic information and has demonstrated an increase in the diagnostic yield by 5-9% [2, 3]. In our cohort, 5.3% of fetuses with normal karyotype results and late-onset fetal abnormalities showed pathogenic CMA results. Furthermore, the CMA and WES results are available in a median of 10 and 20 workdays, respectively. Owing to the higher detection rate, shorter turnaround time, and affordable expense, CMA became the first-tier method in prenatal diagnosis associated with fetal structural anomalies and/or increased nuchal translucency (NT) [1].

Clinical implementation of next-generation sequencing (NGS) in the field of prenatal diagnostics is widely available. Previous studies noted that patients who underwent WES had higher diagnostic yields (25–35%) among fetuses with genetic disorders, while either karyotyping or CMA were negative [4, 5]. Although both WGS and WES can detect novel pathogenic genes, WGS analyzes the entire genome while WES only focuses on the exons [6, 7]. As the exons were demonstrated to have more clinical relevance to human diseases, WES is more frequently used in prenatal diagnosis [6]. Because of the different costs and diagnostic yields of WES and CMA, patients can choose according to clinical geneticists’ suggestion and their financial condition.

Nevertheless, the natural history of several kinds of genetic syndromes necessitates genetic evaluation late in the pregnancy since the evolving abnormal findings can be gradually detected by ultrasound examination [8]. The chances of late-onset abnormalities after 1^st^ and 2^nd^-trimester ultrasound examinations are estimated at 5.5%-17% [9]. A recent study demonstrated that a large number of fetal abnormalities, especially central nervous system (CNS) abnormalities, are detected at 35–37 week scan [8]. Recent studies suggested that late amniocentesis is safe and effective to be performed after 24 weeks onwards [1, 10]. However, a large clinical investigation regarding the indications, procedure-related complications, and pregnancy outcomes of late amniocentesis is lacking. In China, termination of major fetal abnormalities after 24 weeks is legal. This retrospective study aimed to provide more comprehensive clinical data regarding late amniocentesis in prenatal diagnosis.

## Materials and methods

### Data collection

This study was approved by the Research Ethics Committee of the Third Affiliated Hospital of Guangzhou Medical University.

**Fig. 1:**
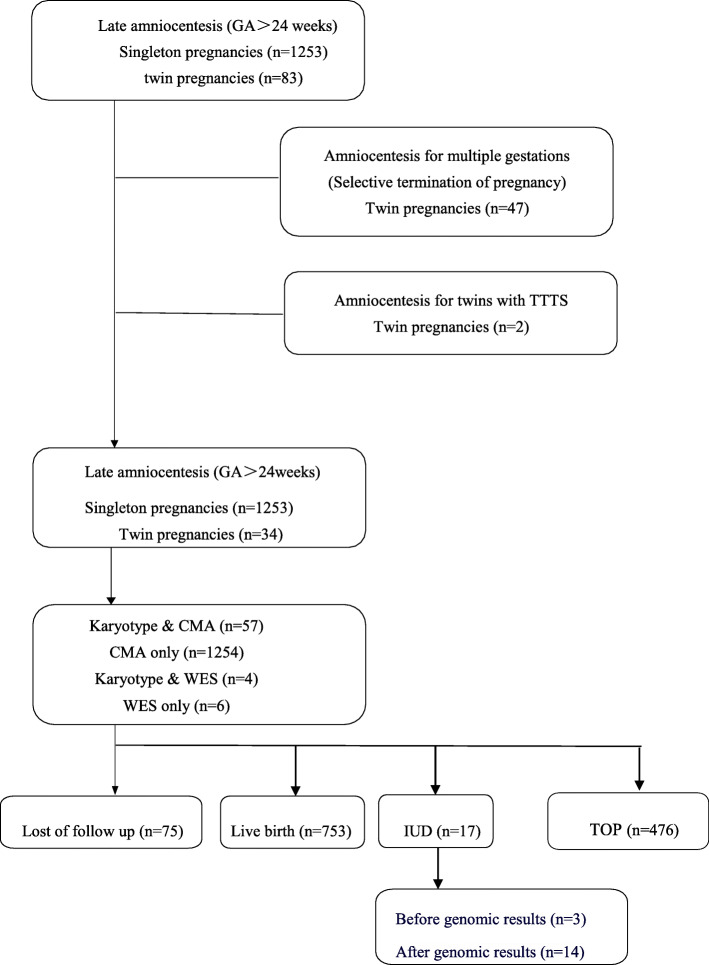
Study population of late amniocentesis. The selection of the study population from all women after 24 gestational weeks in our hospital, who underwent amniocentesis during January 2014 and December 2019

In this study, we conducted a cohort analysis of pregnant women who underwent amniocentesis after 24 weeks in our fetal medicine unit from January 2014 to December 2019. Exclusion criteria were multiple gestations that underwent selective termination of pregnancy and amniocentesis performed for twin to twin transfusion syndrome (TTTS) (Fig. 1).

Patient records were retrieved from the fetal medicine system (Astraia software gmbh, Munich, Germany), including the indications, genetic test results, complications, and pregnancy outcomes which were also obtained by phone contact if the participant did not give birth in our hospital. Information was obtained from case records, validated by research staff, and statistical analyses were performed using IBM SPSS Statistics (version 21; IBM Corporation, Armonk, NY, USA).

### Process of the amniocentesis

All ultrasound scans were performed by FMF certified sonographers, and all patients underwent a pretest and post-test counseling before invasive procedures. The procedures were performed by fetal medicine specialists. A needle puncture was used to get through the patient’s overlying skin into the uterus and amniotic cavity, followed by aspiration of amniotic fluid [11]. The genetic tests were carried out either within our hospital or in the accredited laboratories.

### CMA

Genomic DNA was obtained from amniotic fluid (10 ml) collected by amniocentesis using the QIAamp DNA Mini Kit (Qiagen, Hilden, Germany) according to the manufacturer's instructions. DNA (50 ng) was labeled using Affymetrix Cytogenetics Reagent Kit, and the labeled DNA was applied to an Affymetrix Cytoscan 750K array (Affymetrix Inc., Santa Clara, CA). The platform contains 550,000 non-polymorphic Copy Number Variation (CNV) probes and more than 200,000 Single Nucleotide polymorphism (SNP) probes with an average resolution of 100-kb. Practical procedures were carried out according to the instructions. The data files generated for each sample were analyzed using Chromosome Analysis Suite (ChAS) Software. The characteristics and spectrum of CNV, including the type of aberrations (gains/duplications or losses/deletions), genomic loci, sizes, and the mode of inheritance (familial or de novo) were studied. The data were interpreted by using information available in the scientific literature and public databases (CLIVAR, Database of Genomic Variants, etc.). This information was used to classify detected CNVs based on their expected clinical significance as benign, likely benign, variants of uncertain significance (VOUS), likely pathogenic or pathogenic, following the recommended guidelines from the International Standard Cytogenomic Array and the American College of Medical Genetics (ACMG). Quantitative Fluorescence Polymerase Chain Reaction and multiplex probe ligation assay (MLPA) for common aneuploidies (chromosomes 21, 18, 13, X, and Y) were performed when a rapid result was required. In some cases, with pathognostic ultrasound findings or known family history, targeted fetal molecular diagnosis for specific single-gene mutations was also made [12].

### WES

According to the manufacturer’s instructions, parental blood samples were collected for DNA extraction using the SolPure Blood DNA kit (Magen, Guangzhou, China). The genomic DNA of the fetuses was obtained from the amniotic fluid as described above. The genomic DNA was fragmented by a Q800R Sonicator (Qsonica, Newtown, USA) to generate 300–500 bp DNA fragments. The paired-end libraries were prepared using the library preparation protocol (Illumina, San Diego, CA). Custom designed NimbleGen SeqCap probes (Roche NimbleGen, Madison, WI) were used for in-solution hybridization to enrich target sequences. Genes with the phenotype-causing mutation were identified from Online Mendelian Inheritance in Man (OMIM). Subsequent sequencing of the enriched DNA was performed on a NextSeq500 sequencer (Illumina, San Diego, CA).

Sequencing reads from the fetal DNA were mapped to the reference human genome version hg19 (http://genome.ucsc.edu/). Variants were called and reviewed by NextGENe software (SoftGenetics, State College, PA) and in-house annotation pipeline. Literature, mutation and population databases were used for variant annotation, including 1000 Genomes, dbSNP, GnomAD, Clinvar, HGMD, and OMIM. The synonymous and common SNPs (MAF > 0.1%) were filtered out, and rare variants with high confidence were considered as a disease-causing candidate for further genetic evaluation. Multiple computational algorithms were applied to assist the genetic evaluation of pathogenicity, including SIFT (https://sift.bii.a-star.edu.sg/, Craig Venter Institute), Polyphen-2 (https://genetics.bwh.harvard.edu/pph2/, Harvard University), and Mutation Taster (https://www.mutationtaster.org, NeuroCure Cluster of Excellence). The interpretation of variants was performed according to the ACMG guidelines.

## Results

In our center, women were suggested to undergo amniocentesis, and prenatal genetic test (CMA or WES) when fetal structural anomalies are detected by ultrasound or results of noninvasive prenatal testing (NIPT) indicates high risk of a chromosomal abnormalities.

The demographics data of the late amniocentesis group are given in Fig. 1. Out of the 1336 pregnancies recruited, 1287 were included for analysis, including 1253 singleton pregnancies, 12 monochorionic diamniotic pregnancies, and 22 dichorionic diamniotic twin pregnancies. All the amniocentesis in twin pregnancy were performed with double punctures. Except for 75 women who were lost of follow-up, the pregnancy outcome was available for 1246 fetuses. The median age of the pregnant woman was 29.9 (16.0–46.0) years, and the median gestational age at amniocentesis was 28.0 (24.00–39.57) weeks (Table 1).

**Table 1:** Maternal and fetal characteristics of the late amniocentesis group. The characteristics of the women who underwent late amniocentesis in our hospital during January 2014 and December 2019

Maternal age (years)		29.86 ± 5.58
Gravidity		2 (1–8)
Parity		1 (0–4)
Gestational age at diagnosis (weeks)		30.06 ± 3.84
Gestational age at amniocentesis (weeks)		28.01 ± 3.42
Pregnancy outcome	Live birth	753
	Termination of pregnancy	476
	IUD	17
	Unknown	75

*Abbreviations*: *IUD* Intra uterine death

### Indications

Table 2 showed the indications of late amniocentesis. The most common one was the late detected abnormalities (or a combination of ultrasound abnormalities with other indications) (1130/1321 fetuses, 85.5%). These abnormalities included CNS malformations (250/1321, 18.9%), cardiovascular malformations (177/1321, 13.5%), urinogenital malformations (170/1321, 13.0%) and others. Another common indication of late amniocentesis was positive prenatal screening results (115/1321 fetuses, 8.7%), including abnormal NIPT results and positive second trimester biochemical screening results. Others were abnormal diagnostic test results, advanced maternal age, previous history of pregnancy affected by aneuploidy or genetic disease, and family history of genetic disease.

**Table 2:** Indications for late amniocentesis. Of note, every indication was counted once but many cases had more than one indication.

Indication	Number	Percentage (%)	Pathogenic Results	Diagnostic Yield (%)	VUS
***Late detected abnormalities***	***1130***	***85.5***	***100***	***8.8***	***55***
Central nervous system malformations	250	18.9	15	6.0	14
Cardiovascular malformations	177	13.5	23	13.0	7
Urogenital malformations	170	13.0	11	6.5	12
Multiple malformations	101	7.7	24	23.8	4
Facial malformations	70	5.3	5	7.1	2
Skeletal malformations	68	5.2	4	5.9	3
Polyhydramnios	56	4.3	3	5.4	4
Digestive malformations	55	4.2	0	0	0
SGA	52	4.0	3	5.8	5
FGR	48	3.7	5	10.4	1
Increased NT	25	1.9	5	20.0	1
Fetal tumor	21	1.6	1	4.8	0
Thoracic abnormalities	15	1.1	1	6.7	0
Ascites	10	0.8	0	0	1
Placental anomalies	7	0.5	0	0	0
Oligohydramnios	5	0.4	0	0	1
***Suspected prenatal screening results***	***115***	***8.7***	***32***	***27.8***	***6***
Abnormal NIPT result	84	6.4	30	35.7	5
Positive second trimester DS screening result	31	2.3	2	6.5	1
***Abnormal diagnostic test results***	***10***	***0.8***	***7***	***70***	***0***
Abnormal CMA result	5	0.4	4	80	0
Abnormal karyotyping result	4	0.3	3	75	0
Abnormal FISH result	1	0.1	0	0	0
***Others***	***66***	***5.0***	***2***	***3.0***	***1***
Patient’s request	25	1.9	0	0	1
Advanced maternal age	25	1.9	2	8	0
Abnormal tribunal history	9	0.7	0	0	0
Family history of genetic disease	7	0.5	0	0	0
Total	1321	100	141	10.7	62

*Abbreviations*: *FGR* Fetal growth restriction, defined as an estimated weight less than the 10th percentile [13]; *SGA* Small for gestational age, defined as an estimated weight less than normal fetuses but fail to meet diagnostic requirement for FGR; Polyhydramnios defined as an amniotic fluid index (AFI) > 24 cm or the maximum pool depth (MPD) is > 8 cm; Oligohydramnios defined as a value of AFI < 5 cm or MPD < 2 cm; *NT* Nuchal translucency; Increased NT defined as NT measurement reaches 3.5 mm or more (>99th percentile) [14]; *NIPT* Noninvasive prenatal testing

Table 3 showed the indications of routine amniocentesis. The most common one was fetal abnormalities (979/2177 fetuses, 45.0%) including Increased NT (308/2177, 14.1%), cardiovascular malformations (148/2177, 6.8%), CNS malformations (134/2177, 6.2%) and others.

**Table 3:** Indications for routine amniocentesis. Every indication was counted once but many cases had more than one indication.

Indication	Number	Percentage (%)	Pathogenic Results	Diagnostic Yield (%)	VUS
***Fetal abnormalities***	***979***	***45.0***	***125***	***12.8***	***35***
Increased NT	308	14.1	43	14.0	8
Cardiovascular malformations	148	6.8	19	12.8	8
Central nervous system malformations	134	6.2	11	8.2	3
Multiple malformations	96	4.4	32	33.3	3
Urogenital malformations	81	3.7	5	6.2	5
Facial malformations	66	3.0	4	6.1	3
Skeletal malformations	47	2.2	2	4.3	0
Digestive malformations	21	1.0	1	4.8	0
FGR	20	0.9	1	5.0	0
Fetal tumor	19	0.9	4	21.1	1
Chest malformations	16	0.7	2	12.5	0
Polyhydramnios	8	0.4	1	12.5	0
Oligohydramnios	6	0.3	0	0	0
Ascites	5	0.2	0	0	0
Placental anomalies	4	0.2	0	0	4
***Suspected prenatal screening results***	***612***	***28.1***	***177***	***28.9***	***26***
Abnormal NIPT result	436	20.0	167	38.3	19
Positive second trimester DS screening result	176	8.1	10	5.7	7
***Abnormal diagnostic test results***	***36***	***1.7***	***9***	***25***	***2***
Abnormal CMA result	21	1.0	6	28.6	1
Abnormal karyotyping result	15	0.7	3	20.0	1
***Others***	***549***	***25.2***	***23***	***4.2***	***26***
Abnormal tribunal history	188	8.6	8	4.3	8
Advanced maternal age	163	7.5	8	4.9	8
Family history of genetic disease	102	4.7	3	2.9	6
Patient’s request	96	4.4	4	4.2	4
Total	2177	100	334	15.3	89

*Abbreviations*: *FGR* Fetal growth restriction, defined as an estimated weight less than the 10th percentile [13]; Polyhydramnios defined as an amniotic fluid index (AFI) > 24 cm or the maximum pool depth (MPD) is > 8 cm; Oligohydramnios defined as a value of AFI < 5 cm or MPD < 2 cm; *NT* Nuchal translucency; Increased NT defined as NT measurement reaches 3.5 mm or more (>99th percentile) [14]; *NIPT* Noninvasive prenatal testing

### Complications

In the late amniocentesis group, only one chorioamnionitis was identified on the third day after the amniocentesis. A total of 33 preterm birth (PTB) (33/1321, 2.5%) and 17 intrauterine death (IUD) (17/1321, 1.3%) were identified. Six PTBs (6/33, 18.2%) and 3 IUDs (3/17, 17.6%) occurred within the first week after amniocentesis. Five PTBs (5/33, 15.2%) and 5 IUDs (5/17, 29.4%) developed after the first week but within one month after amniocentesis. The remaining PTBs (22/33, 66.7%) and IUDs (9/17, 52.9%) cases happened after one month of the amniocentesis. The overall complication rate was 3.9% (51/1321), and 80.4% (41/51) of the complications are associated with fetal abnormalities, and 7.8% (4/51) of them happened in fetuses with abnormal NIPT results (Table S1). In the routine amniocentesis group, the overall complication rate was 4.0% (88/2177), including 39 PTBs (39/2177, 1.8%) and 49 IUDs (49/2177, 2.3%) (Table S3). There was no significant difference between the two groups (*p* = 0.43).

Among women who underwent late amniocentesis, PTBs occurred in 0.3% (4/1253) and 0.4% (5/1253) in singleton pregnancies, while that happened in 5.9% (2/34) and 0% in twin pregnancies (within one week and one-month post-procedure, respectively). The earliest PTB happened in a singleton pregnancy on the third day after the procedure. Ninety-one percentage of PTBs (30/33) occurred in fetuses with ultrasound anomalies. Women with age < 35 years and ≥ 35 years had a PTB rate of 2.4% (25/1024) and 2.9% (8/273), respectively.

IUD took place in 0.9% (12/1253) singleton pregnancies and 8.8% (3/34) twin pregnancies. Twenty-nine percentage (5/17) of the IUDs are pathogenic chromosomal disorders. The earliest IUD took place in a singleton pregnancy with CNS malformations one day after the procedure. Out of the 17 IUDs, there were four fetal growth restriction (FGR) [13], three CNS malformations, two cardiovascular malformations, two skeletal malformations, two multiple malformations, one urogenital malformation, and three structurally normal fetuses. It should be noted that three of the 17 IUDs cases without fetal malformations are a fetus with abnormal CMA result and twins with abnormal NIPT results. In addition, two pairs of DCDA suffered from IUD within one month after the procedure. One of the MCDA suffered from IUD after one month, and another was live birth.

### Pathogenic findings

#### CMA

CMA was performed in 1311 fetuses in the late amniocentesis group, and chromosomal disorders were identified in 141 (141/1311, 10.8%) of them. Sixty-nine were aneuploidies (69/1311, 5.3%), including thirty-eight trisomy 21, nine trisomy 18, and five trisomy 13. Other aneuploidies included sex chromosomal abnormality (like XXX, XXY, and XYY), trisomy 8, trisomy 9, and trisomy 12 (Table S2). Pathogenic copy number abnormalities were identified in 72 (72/1311, 5.5%) fetuses by CMA. Karyotyping was performed in 23 of them. Only three CNVs (3/23, 13.0%) could be correctly detected via karyotyping (a deletion on chr18q22.3q23, a deletion on chr4p16.3p15.2, and a duplication on 5q21.1q22.2). The other 20 CNVs (20/23, 87.0%) detected by CMA could not be identified via karyotyping. Uncertain results were reported in sixty-three cases (63/1311, 4.8%), including 51 VUS results, seven likely pathogenic results, and five likely benign results.

In the subgroup of fetuses with abnormalities, the diagnostic rate of CMA was 8.9% (101/1131). Only 41 fetuses (41/141) with pathogenic CMA results were not associated with structural abnormalities. Furthermore, compared with fetuses with isolated abnormalities (76/1029, 7.4%), fetuses with multiple abnormalities achieved a higher positive yield (24/101, 23.8%).

In the routine amniocentesis group, chromosomal abnormalities were identified in 335 (335/2177, 15.4%) of them, and 12.9% of them were fetuses with structural abnormalities (Table S4).

#### WES

WES was carried out in ten families (parents and fetuses). Chromosomal abnormalities were identified in one fetus (1/10, 10%). Two copy number variations were considered to be likely pathogenic (2/10, 20%), while another two cases were likely benign (2/10, 20%).

Karyotyping had performed in 4 of 10 cases simultaneously, but two of them (one likely benign and one likely pathogenic) showed different results from WES.

The ultrasound findings supported two WES reports. In the first case, the pregnant woman with pathogenic WES underwent amniocentesis at 27 weeks due to FGR identified by ultrasound. Trio-exome sequencing showed a mutation c.625 + 1G > A in the SLC7A7 gene compatible with fetal Lysinuric protein intolerance (LPI). The couple decided to continue the pregnancy, but IUD took place two months after the amniocentesis. In the second case, the couple was referred for genetic counseling in their fourth pregnancy due to fetal encephalocele, hydrocephalus, and cerebellum dysplasia detected at 25 weeks. WES reported ISPD gene mutations, c.674delC (p.A225Dfs*21) and c.1106 T > G (p.V369G). It was consistent with Walker-Warburg syndrome. They finally decided to terminate the pregnancy.

#### Pregnancy outcome

The median turnaround time for receipt of CMA and WES results were 10 and 20 workdays, respectively. Except for nine patients suffering from complications within one week after amniocentesis, all other pregnant women (1278/1287, 99.3%) received their genetic report before delivery or termination of pregnancies (Table S1).

A total of 753 (751/1321, 56.9%) pregnancies resulted in live births, 476 (476/1321, 36.0%) pregnancies were terminated, 17 (17/1321, 1.3%) fetuses died in utero, and 75 (75/1321, 5.7%) cases were lost of follow up.

Among the 476 terminated pregnancies, pathogenic results were reported in 124 cases (124/476, 26.1%). Except for 74 cases that were lost of follow up, six PTB happened before patients received the results, 12 fetuses (12/112, 31.1%) died in uterus, and 712 fetuses were live birth, a total of 324 couples terminated the pregnancy despite the normal genomic results (324/1123, 28.9%) because of the fetal abnormalities detected by ultrasound, especially urinogenital and cardiovascular malformations.

Thirty-one of 69 (44.9%) fetuses diagnosed with a VUS, likely pathogenic or likely benign by CMA or WES in the report. The couples decided to terminate the pregnancy due to the abnormal finding on ultrasound examination. Others were live birth, except one was lost of follow up, and the other two were IUD. In addition, 87.3% (124/142) of the fetuses with pathogenic results were terminated. In 69 fetuses with aneuploidy identified by CMA, there were 34 out of 38 pregnancies with trisomy 21 two IUD), 9 out of 9 pregnancies with trisomy 18, 5 out of 5 pregnancies with trisomy 13, and 14 out of 17 with other aneuploidy were terminated after receiving the genetic reports. The remain five in the 69 women decided to give birth to the babies. Sixty-one out of 73 pregnancies with pathogenic CNVs identified by CMA or WES were terminated. Two fetuses with CNVs died, and another one was associated with PTB before receiving the genetic result. Except one was lost, the other seven women decided to continue the pregnancy and gave birth to the babies.

## Discussion

The commonest indication of routine amniocentesis (16–24 weeks) in our cohort is abnormal NIPT results and the increased NT [14]. However, late detected fetal abnormalities (or a combination of fetal abnormalities with other indications) are the most common indication of late amniocentesis, accounted for 85.5% in our cohort, consistent with a recent study [8]. The central nervous system abnormalities (184/1130, 16.3%) was the most common ones.

Our sample size is larger than the other studies. The overall complication rate after late amniocentesis is 3.9%. Although it is higher than that reported by Liao et al. (1.9%) [15], the overall complication rate in our cohort is lower than that reported by Daum et al. (6.2%) [1], Geffen et al. (6.6%) [10], Gabbay et al. (8%) [16] and a recent meta-analysis [17]. In our cohort, there was no significant difference between the overall complication rate of the late amniocentesis group and the routine group (*p* = 0.43), and it is reasonable to speculate that at least some of the complications of late amniocentesis are unlikely to have a direct association with amniocentesis because 80.4% (41/51) complications took place in fetuses with abnormalities.

We did not analyze all kinds of complications but focused on PTB and IUD, which were the most common ones. PTB happened in 33 women (2.5%) after amniocentesis, of which four had pathogenic chromosomal disorders, whereas five of seventeen women suffered from IUD (1.3%) had pathogenic chromosomal disorders. In our cohort, PTBs were associated with fetal malformations (25/33, 75.8%), mainly CNS malformations (5/25, 20%), and FGR (4/25, 16%). PTB happened in 0.7% (9/1253) in singleton pregnancies, while it occurred in 5.9% (2/34) in twin pregnancies within one-month post-procedure. IUD occurred in 0.3% in singleton pregnancies, while it happened ed. in 5.9% in twin pregnancies within one-month post-procedure. Only three of the 17 IUD cases reported no fetal malformations. Only one chorioamnionitis was identified in the late amniocentesis group after the amniocentesis, and no chorioamnionitis was found in the routine group.

The presence of fetal malformations and pathogenic chromosomal disorders obviously increases the risk of both PTB and IUD, compared to others without these risk factors. In addition, twin pregnancies are more likely to be high-risk for PTB and IUD after late amniocentesis. This result may provide clinicians information to balance both the indication for late amniocentesis and the risks.

The total yield of abnormal genetic results for late amniocentesis was 10.7%, which was higher than a recent study by Geffen et al. [10] and Daum et al. [1] with similar indications, and is a comparable rate to that of routine amniocentesis (15.4%). As a referral center, a significant number of our patients are referred due to guarded prognosis. However, 86.4% of pathogenic CNVs failed to be detected by karyotyping, demonstrated that both CMA and WES achieve better diagnostic yield than traditional karyotyping as that in the second-trimester amniocentesis [2, 9]. In the late amniocentesis group, the diagnostic rate reaches the highest (70.0%) when abnormal diagnostic test results become the indication of amniocentesis, following by abnormal NIPT results (35.7%) and it is comparable with the diagnostic rate of the routine amniocentesis for abnormal NIPT results (168/436, 38.5%). Most women (87.3%) decided to terminate the pregnancies after receiving pathogenic genetic results. Twenty-nine percent of women with normal genetic results still opted for termination of pregnancies due to the severe ultrasound findings.

In China, termination of pregnancies after 24 weeks is legal in cases with major fetal abnormalities. Therefore, late amniocentesis can provide more information for patients to decide whether to continue the pregnancy. Even in places where late termination is not allowed, performing late amniocentesis and exploring the etiology remains useful. It can provide patients opportunities to begin to anticipate lifestyle changes and apply for assistance from relevant supporting groups and resources.

A considerable disadvantage of late amniocentesis, is the identification of uncertain results like VUS, likely pathogenic and likely benign. Forty-five percentage of fetuses with uncertain results were terminated. The reason is that 88.7% of them are with fetal abnormalities, while the other 12.3% are fetuses with abnormal NIPT results. Except one patient is lost, and the other two died, half of the eight fetuses with pathogenic or likely pathogenic results are terminated regardless of the abnormal ultrasound finding.

Pregnant women paid close attention to their genetic test results, which made it reasonable to consider the importance and necessity of genetic information even if ultrasound findings are severely abnormal. Moreover, genetic information also plays an important role in future pregnancies. To increase the accuracy of diagnosis and save time, we also recommend offering CMA and WES sequentially when sonographic abnormalities were identified. However, cost-effectiveness should be assessed.

### Strengths and limitations

Our study has some limitations. There were 75 women lost during the follow-up. Our study started in 2014. However, WES was underdeveloped and unavailable at that time. All our cases of WES were collected in the last two years. Furthermore, we did not analyze all kinds of complications but only focused on PTB and IUD, which were most common. The assessment of complications and diagnostic yield could be inadequate. In addition, our institution is a tertiary referral center. Patients suggestive of abnormalities may have been preferentially referred to our department, causing a bias in patient selection.

This study also has several strengths. Although previous studies [1, 10, 15] have reported some data of late amniocentesis, the sample size of our cohort is larger than the previous ones so that our data could be more persuasive. Our study provided information on late amniocentesis, which can improve the prenatal diagnosis and postnatal care for women.

## Conclusion

Late amniocentesis is a reasonable procedure in modern genetic technologies when late-onset sonographic abnormalities are identified. It is a quick and helpful tool for pregnant women after 24 gestational weeks. The majority of patients can get their genetic results before delivery, and enough time would be provided to make a decision about the pregnancy. The diagnostic yields of CMA and WES are much higher than that of traditional karyotype. In cases with normal karyotype results and sonographic abnormalities, CMA and WES should be considered.

The diagnostic yield achieved maximal when fetuses with suspected genetic disorders become the indication of late amniocentesis, following by sonographic abnormalities. The risk of PTB and IUD should be considered with the presence of sonographic abnormalities.

## Supplementary Information

**Additional file 1.**

## Data Availability

The dataset generated and/or analyzed during this study are available from the corresponding author on reasonable request.

## Abbreviations

CMA: Chromosomal microarray analysis
CNS: Central nervous system
CNV: Copy number variants
DCDA: Dichorionic diamniotic twin pregnancies
FGR: Fetal growth restriction
IUD: Intra uterine death
MCDA: Monochorionic diamniotic pregnancies
NIPT: Noninvasive prenatal testing
NT: Nuchal translucency
PTB: Preterm birth
TTTS: Twin to twin transfusion syndrome
VUS: Variants of uncertain significance
WES: Whole-exome sequencing
WGS: Whole-genome sequencing
