# Supplementary material for: The application of late amniocentesis: a retrospective study in a tertiary fetal medicine center in China
Source: BMC Pregnancy Childbirth. 2021 Mar 30;21:266. doi: 10.1186/s12884-021-03723-7 (PMC8011189; doi:10.1186/s12884-021-03723-7)
Supplement: Supplementary file 1 — Additional file 1. [file 12884_2021_3723_MOESM1_ESM.docx]

**Table S1 Summary of the complications of late amniocentesis**

| Complication | Amniocentesis to Complication interval | Gestation | Age | Timing of amniocentesis | Indication of  amniocentesis | Genomic  result | | Genomic results  before delivery  (Y/N) | Fetal  outcome |
| --- | --- | --- | --- | --- | --- | --- | --- | --- | --- |
| **PTB**  **(33)** | Within one week  (6) | Singleton (4) | 38 | 35+3 | Digestive malformations | | Normal | N | LB |
|  |  |  | 40 | 27+4 | FGR | | Normal | N | LB |
|  |  |  | 26 | 32+2 | Placental anomalies | | Normal | N | LB |
|  |  |  | 32 | 35+5 | Skeletal malformations | | CNV | N | LB |
|  |  | MCDA (1) | 30 | 32+4 | Multiple malformations | | Normal | N | LB |
|  |  |  |  |  | Normal | | Normal | N | LB |
|  |  | DCDA (1) | 39 | 32+0 | Multiple malformations | | Normal | N | LB |
|  |  |  |  |  | CNS malformations | | T21 | N | LB |
|  | Within one month  (5) | Singleton (5) | 33 | 27+4 | CNS malformations | | CNV | Y | LB |
|  |  |  | 31 | 31+0 | Digestive malformations | | Normal | Y | LB |
|  |  |  | 29 | 31+0 | Cardiovascular malformations | | Normal | Y | LB |
|  |  |  | 24 | 33+0 | CNS malformations | | Normal | Y | LB |
|  |  |  | 31 | 29+0 | Polyhydramnios | | Normal | Y | LB |
|  | After one month  (22) | Singleton (16) | 27 | 26+5 | Cardiovascular malformations | | Normal | Y | LB |
|  |  |  | 21 | 24+6 | Facial malformations | | Normal | Y | LB |
|  |  |  | 44 | 31+1 | FGR | | CNV | Y | LB |
|  |  |  | 21 | 30+5 | Multiple malformations | | Normal | Y | LB |
|  |  |  | 34 | 27+0 | SGA | | Normal | Y | LB |
|  |  |  | 24 | 29+5 | Cardiovascular malformations | | Normal | Y | LB |
|  |  |  | 29 | 31+6 | SGA | | Normal | Y | LB |
|  |  |  | 35 | 27+0 | Family history of genetic disease | | Normal | Y | LB |
|  |  |  | 25 | 26+3 | Placental anomalies | | Normal | Y | LB |
|  |  |  | 30 | 27+3 | FGR | | Normal | Y | LB |
|  |  |  | 38 | 32+0 | Urogenital malformations | | Normal | Y | LB |
|  |  |  | 39 | 24+4 | FGR | | Normal | Y | LB |
|  |  |  | 27 | 29+1 | CNS malformations | | Likely benign | Y | LB |
|  |  |  | 29 | 26+0 | Urogenital malformations | | Likely pathogenic | Y | LB |
|  |  |  | 27 | 29+3 | Digestive malformations | | Normal | Y | LB |
|  |  |  | 35 | 27+0 | CNS malformations | | Normal | Y | LB |
|  |  | MCDA (2) | 28 | 25+3 | CNS malformations | | Normal | Y | LB |
|  |  |  |  |  | CNS malformations | | Normal | Y | LB |
|  |  |  | 34 | 28+0 | Abnormal NIPT | | Normal | Y | LB |
|  |  |  |  |  | Abnormal NIPT | | Normal | Y | LB |
|  |  | DCDA (4) | 36 | 24+0 | Cardiovascular malformations | | Normal | Y | LB |
|  |  |  |  |  | Normal | | Normal | Y | LB |
|  |  |  | 34 | 24+2 | CNS malformations | | Normal | Y | LB |
|  |  |  |  |  | Normal | | Normal | Y | LB |
|  |  |  | 30 | 25+4 | Cardiovascular malformations | | Normal | Y | LB |
|  |  |  |  |  | Normal | | Normal | Y | LB |
|  |  |  | 25 | 24+0 | Skeletal malformations | | Normal | Y | LB |
|  |  |  |  |  | Normal | | Normal | Y | LB |
| **IUD**  **(17)** | Within one week  (3) | Singleton (3) | 21 | 37+5 | CNS malformations | | Normal | N | IUD |
|  |  |  | 24 | 32+2 | Abnormal CMA | | T21 | N | IUD |
|  |  |  | 25 | 24+6 | CNS malformations | | CNV | N | IUD |
|  | Within one month  (5) | Singleton | 31 | 31+6 | Urogenital malformations | | Normal | Y | IUD |
|  |  | DCDA（2） | 32 | 24+5 | Abnormal NIPT | | T21 | Y | IUD |
|  |  |  |  |  | Abnormal NIPT | | Normal | Y | IUD |
|  |  |  | 29 | 32+0 | Skeletal malformations | | Normal | Y | IUD |
|  |  |  |  |  | Multiple malformations | | VUS | Y | IUD |
|  | After one month  (9) | Singleton (8) | 26 | 24+0 | Cardiovascular malformations | | Normal | Y | IUD |
|  |  |  | 41 | 27+0 | FGR | | CNV | Y | IUD |
|  |  |  | 36 | 30+5 | CNS malformations | | VUS | Y | IUD |
|  |  |  | 31 | 29+0 | FGR | | Normal | Y | IUD |
|  |  |  | 38 | 27+0 | Skeletal malformations | | Normal | Y | IUD |
|  |  |  | 28 | 25+2 | FGR | | Normal | Y | IUD |
|  |  |  | 31 | 25+5 | Multiple malformations | | Normal | Y | IUD |
|  |  |  | 24 | 26+0 | Cardiovascular malformations | | Normal | Y | IUD |
|  |  | MCDA | 32 | 25+1 | FGR | | CNV | Y | IUD |
|  |  |  |  |  | Normal | | Normal | Y | LB |
| **Chorioamnionitis** | Three days | Singleton | 28 | 28+0 | Multiple malformations | | Normal | Y | TOP |
| Total | 51 |  |  |  |  | |  |  |  |

Abbreviations: PTB-Preterm birth; IUD-Intra uterine death; MCDA-Monochorionic diamniotic pregnancies with double puncture; DCDA-Dichorionic diamniotic twin pregnancies with double puncture; FGR-Fetal growth restriction; SGA-Small for gestational age infant; NIPT-Noninvasive prenatal testing; CMA-Chromosomal microarray analysis; CNS malformations-Central nervous system malformations; VUS-Variants of uncertain significance; LB-Live birth; TOP-Termination of the pregnancy.

**Table S2 CMA results of late amniocentesis**

| CMA results | Details | Maternal  Age | Timing of amniocentesis | Indication of  amniocentesis | Karyotyping  (-/results) | Fetal  outcome |
| --- | --- | --- | --- | --- | --- | --- |
| ***pathogenic*** | ***141*** |  |  |  |  |  |
| Aneuploidies  (69) | Trisomy 21（38） | 36 | 24+0 | Urogenital malformations | - | TOP |
|  |  | 37 | 25+0 | Cardiovascular malformations | T21 | TOP |
|  |  | 26 | 25+1 | Cardiovascular malformations | - | TOP |
|  |  | 18 | 31+6 | Multiple malformations | - | TOP |
|  |  | 26 | 25+0 | Multiple malformations | - | TOP |
|  |  | 25 | 24+0 | Cardiovascular malformations | - | TOP |
|  |  | 42 | 27+0 | Abnormal FISH | - | TOP |
|  |  | 24 | 32+2 | Abnormal CMA | - | IUD |
|  |  | 35 | 36+0 | Abnormal NIPT | T21 | TOP |
|  |  | 34 | 33+6 | Multiple malformations | T21 | TOP |
|  |  | 32 | 24+5 | Abnormal NIPT | - | IUD |
|  |  | 29 | 24+0 | Increased NT | T21 | TOP |
|  |  | 29 | 25+0 | Cardiovascular malformations | - | TOP |
|  |  | 46 | 32+1 | Polyhydramnios | - | TOP |
|  |  | 23 | 25+0 | Abnormal NIPT | - | TOP |
|  |  | 30 | 24+0 | CNS malformations | - | TOP |
|  |  | 26 | 31+5 | CNS malformations | - | TOP |
|  |  | 36 | 28+0 | Abnormal NIPT | - | TOP |
|  |  | 37 | 24+0 | CNS malformations | - | TOP |
|  |  | 40 | 25+2 | Abnormal NIPT | T21 | LB |
|  |  | 39 | 28+0 | Increased NT | - | TOP |
|  |  | 40 | 31+0 | Abnormal NIPT | T21 | TOP |
|  |  | 39 | 32+0 | CNS malformations | T21 | LB |
|  |  | 27 | 24+0 | CNS malformations | - | TOP |
|  |  | 31 | 30+0 | Abnormal NIPT | - | TOP |
|  |  | 44 | 29+0 | Cardiovascular malformations | - | TOP |
|  |  | 42 | 25+0 | Abnormal NIPT | - | TOP |
|  |  | 31 | 25+0 | Urogenital malformations | - | TOP |
|  |  | 39 | 36+4 | Facial malformations | - | TOP |
|  |  | 46 | 24+0 | Advanced maternal age | - | TOP |
|  |  | 42 | 24+1 | Increased NT | - | TOP |
|  |  | 33 | 24+0 | Abnormal NIPT | - | TOP |
|  |  | 34 | 30+0 | FGR | - | TOP |
|  |  | 29 | 26+4 | Abnormal NIPT | - | TOP |
|  |  | 27 | 28+5 | Abnormal NIPT | - | TOP |
|  |  | 24 | 26+1 | Skeletal malformations | - | TOP |
|  |  | 26 | 32+4 | Abnormal NIPT | - | TOP |
|  |  | 27 | 25+3 | Abnormal NIPT | - | TOP |
|  | Trisomy 18（9） | 39 | 33+0 | Multiple malformations | T18 | TOP |
|  |  | 34 | 27+0 | Multiple malformations | T18 | TOP |
|  |  | 28 | 24+4 | Cardiovascular malformations | T18 | TOP |
|  |  | 33 | 25+0 | Multiple malformations | - | TOP |
|  |  | 23 | 32+0 | Abnormal NIPT | T18 | TOP |
|  |  | 39 | 25+6 | Multiple malformations | T18 | TOP |
|  |  | 39 | 24+0 | Abnormal NIPT | T18 | TOP |
|  |  | 46 | 26+0 | Multiple malformations | - | TOP |
|  |  | 33 | 26+0 | Multiple malformations | T18 | TOP |
|  | Trisomy 13（5） | 33 | 25+6 | Multiple malformations | T13 | TOP |
|  |  | 33 | 27+0 | Abnormal NIPT | T13 | TOP |
|  |  | 36 | 25+4 | Facial malformations | - | TOP |
|  |  | 21 | 26+0 | Cardiovascular malformations | - | TOP |
|  |  | 30 | 24+1 | Multiple malformations | T13 | TOP |
|  | Trisomy 8 | 30 | 26+0 | Urogenital malformations | - | TOP |
|  | Trisomy 9 | 29 | 30+5 | Cardiovascular malformations | - | TOP |
|  | Trisomy 12 | 42 | 24+3 | Multiple malformations | - | TOP |
|  | XYY | 27 | 25+0 | Increased NT | XXY | TOP |
|  | XO（4） | 29 | 24+0 | Fetal tumor | XO | TOP |
|  |  | 30 | 26+0 | Increased NT | XO | TOP |
|  |  | 38 | 26+5 | Abnormal CMA | XO | LB |
|  |  | 20 | 24+6 | Cardiovascular malformations | - | TOP |
|  | XXX（4） | 41 | 24+1 | Abnormal NIPT | XXX | LB |
|  |  | 30 | 24+0 | Abnormal NIPT | XXX | TOP |
|  |  | 32 | 26+0 | Abnormal NIPT | - | TOP |
|  |  | 44 | 33+0 | Abnormal NIPT | - | LB |
|  | XXY (5) | 29 | 25+0 | Abnormal NIPT | XYY | TOP |
|  |  | 39 | 24+2 | Abnormal NIPT | XYY | TOP |
|  |  | 30 | 26+6 | Abnormal NIPT | - | TOP |
|  |  | 23 | 27+0 | Abnormal NIPT | XYY | TOP |
|  |  | 30 | 24+0 | Abnormal NIPT | - | TOP |
| CNV  (72) | arr[hg19]Xp22.31 (6,455,151-8,141,076)×1 | 24 | 30+0 | CNS malformations | - | TOP |
|  | arr[hg19]22q11.21(18,631,364-20,312,661) x1; arr[hg19]16p11.2(29,591,326-30,176,508) x3 | 38 | 29+0 | Polyhydramnios | - | TOP |
|  | 4q35.1q35.2(186,150,234-189,539,349) x1 | 31 | 31+1 | Abnormal karyotyping result | - | TOP |
|  | arr[hg19]17p12p11.2(15,162,475-18,922,171) x3; arr[hg19]20p13p11.1(61,661-25,969,009) x3; arr[hg19]20q13.31q13.33(55,167,384-62,913,645) x3; | 28 | 35+0 | Polyhydramnios | Normal | TOP |
|  | Xp deletion | 24 | 31+0 | Multiple malformations | - | TOP |
|  | arr[hg19]22q11.21(18,909,032-21,357,982) ×1 | 24 | 31+0 | CNS malformations | Normal | TOP |
|  | arr[hg19]9p24.3p23(208,454-9,893,613) x1 | 36 | 25+1 | Cardiovascular malformations | - | TOP |
|  | arr[hg19]6q27(165,051,708-170,914,297) x1，arr[hg19]Xp22.33p22.31(168,551-7,432,529) x3 | 33 | 27+4 | CNS malformations | - | PTB |
|  | arr[hg19]13q12.3q34(31,140,327-115,107,733) x3 | 35 | 29+0 | Urogenital malformations | T13 | TOP |
|  | arr[hg19]Yp11.31p11.2(2,650,424-9,138,568) x2，arr[hg19]Yp11.2q11.221(9,167,317-16,963,638) x2 | 38 | 25+2 | Abnormal karyotyping result | - | TOP |
|  | arr[hg19]17q24.2q25.3(66,500,116-81,041,823) x3 | 24 | 32+4 | Multiple malformations | Normal | TOP |
|  | arr[hg19]11q23.3q25(116,683,754-134,937,416) x3，arr[hg19]22q11.1q11.21(16,888,899-20,312,661) x3 | 30 | 24+0 | CNS malformations | Normal | TOP |
|  | arr[hg19]9p24.3q33.1(208,454-121,129,461) x3，arr[hg19]15q11.2(23,021,272-23,625,785) x1， arr[hg19]Yq11.223(24,216,828-24,985,599) x0 | 20 | 26+0 | CNS malformations | Normal | TOP |
|  | arr[hg19]11q23.3q25(119,976,308-134,937,416) x1 | 22 | 25+0 | Cardiovascular malformations | Normal | TOP |
|  | arr[hg19]11q23.3q25(119,976,308-134,937,416) x1 | 34 | 28+0 | Cardiovascular malformations | Normal | TOP |
|  | 8p23.3p23.2(158,048-2,330,754) x1 | 37 | 25+0 | Skeletal malformations | Normal | TOP |
|  | 5q21.1q22.2(100,985,750-111,944,682) x3 | 37 | 28+0 | Abnormal NIPT | 46XN, dup (5) (q14q15) | TOP |
|  | arr[hg19]6q16.1q22.33(98,351,396-127,801,511) x1 | 27 | 28+4 | Cardiovascular malformations | - | TOP |
|  | arr[hg19]17q11.2(28,964,063-30,341,286) x3 | 21 | 36+5 | Multiple malformations | Normal | TOP |
|  | arr[hg19]18q22.3q23(72,905,846-78,013,728) x1 | 40 | 25+6 | Advanced maternal age | 45,XN，der(18;22)(q23;q11) | TOP |
|  | arr[hg19]1q43q44(238,904,667-249,224,684) x1，arr[hg19]18q12.3q23(42,711,437-78,013,728) x3 | 25 | 24+6 | CNS malformations | - | IUD |
|  | arr[hg19]4p16.3p15.2(68,345-21,759,310) x1 | 37 | 24+0 | Abnormal NIPT | 46，？del（4）（p15） | TOP |
|  | arr[hg19]8p23.3q11.21(158,048-50,023,568) x3 | 40 | 25+3 | Multiple malformations | - | TOP |
|  | arr[hg19]22q11.21(19,024,793-21,800,471) x1 | 26 | 30+5 | Multiple malformations | Normal | TOP |
|  | arr[hg19]12p12.1p11.1(25,600,696-34,435,128) x1 | 31 | 26+0 | FGR | - | TOP |
|  | arr[hg19]1q42.3q44(236,509,301-249,224,684) x1，arr[hg19]13q31.3q34(93,051,804-115,107,733) x3，arr[hg19]12p13.33p12.3(173,786-19,752,605) x3，arr[hg19]18q12.1q23(29,208,109-78,013,728) x3 | 25 | 33+3 | Multiple malformations | - | TOP |
|  | arr[hg19]4q34.1q35.2(174,919,364-190,957,460) x1 | 30 | 25+2 | Cardiovascular malformations | - | TOP |
|  | arr[hg19]2q21.2q32.1 (135,005,276-187,747,001) x3 | 24 | 28+2 | Multiple malformations | Normal | TOP |
|  | arr[hg19]7p22.3p21.1(43,376-17,430,718) x3，arr[hg19]8p23.3p23.2(158,048-4,146,842) x1 | 37 | 24+2 | CNS malformations | - | TOP |
|  | arr[hg19]18p11.32p11.21(136,227-14,966,334) x1 | 39 | 27+0 | Abnormal karyotyping result | - | TOP |
|  | arr[hg19]22q11.21(18,648,855-21,800,471) x1 | 27 | 24+0 | Cardiovascular malformations | - | TOP |
|  | arr[hg19]22q11.21q11.22(21,464,763-22,962,962) x1 | 25 | 32+5 | Urogenital malformations | Normal | LB |
|  | arr[hg19]7q11.23(72,669,480-74,146,927) x1 | 29 | 32+4 | Cardiovascular malformations | - | TOP |
|  | arr[hg19]1p36.33p36.31(849,466-7,042,183) x1 | 29 | 28+4 | CNS malformations | Normal | Lost |
|  | arr[hg19]Xp22.31(6,455,151-8,144,378) x1 | 25 | 27+1 | CNS malformations | - | TOP |
|  | MOS dup 4q，arr[hg19]Xp22.33q28(168,551-155,233,098) x1-2 | 28 | 32+3 | Multiple malformations | - | TOP |
|  | arr[hg19]7p14.1(40,748,621-42,108,287) x1 | 26 | 24+0 | Cardiovascular malformations | - | LB |
|  | arr[hg19]4p16.3p16.1(68,345-8,037,418) x1， arr[hg19]4p16.1p15.33(8,043,770-13,253,562) x3 | 21 | 33+5 | SGA | - | TOP |
|  | arr[hg19]6q24.2q25.1(144,902,123-150,394,779) x1 | 33 | 25+0 | Cardiovascular malformations | - | TOP |
|  | arr[hg19]21q11.2q21.2(15,016,486-24,118,504) x4 | 40 | 31+0 | Urogenital malformations | - | LB |
|  | arr[hg19]3q28(188,465,893-189,665,334) x1 | 33 | 25+0 | Facial malformations | Normal | TOP |
|  | arr[hg19]18q23(73,830,798-78,013,728) x3 |  | 30+0 | Abnormal CMA | - | TOP |
|  | arr[hg19]Xp22.33p22.31(168,551-9,355,864) x0 dn | 22 | 26+0 | CNS malformations | - | TOP |
|  | arr[hg19]7p22.3p22.1(43,376-5,893,763) x3， arr[hg19]18q22.1q23(65,027,042-78,013,728) x1 | 33 | 27+3 | CNS malformations | - | TOP |
|  | arr[hg19]22q11.21(18,648,855-21,800,471) x1 | 34 | 26+3 | Multiple malformations | - | TOP |
|  | arr[hg19]22q11.21(18,648,855-21,800,471) x1 | 29 | 26+0 | Cardiovascular malformations | - | TOP |
|  | arr[hg19]22q11.21(18,648,855-21,800,471) x1 | 35 | 24+0 | Urogenital malformations | Normal | TOP |
|  | arr[hg19]4p16.3p16.1(68,345-8,530,222) x1 | 32 | 24+0 | Cardiovascular malformations | Normal | TOP |
|  | arr[hg19]15q11.2q13.3(22,770,421-32,439,524) x4 | 32 | 35+5 | Skeletal malformations | - | PTB |
|  | arr[hg19]22q11.21(18,919,477-21,915,207) x3 | 20 | 24+5 | Cardiovascular malformations | - | LB |
|  | MOS LOH 1qter | 23 | 34+0 | Positive second trimester DS screening result | - | TOP |
|  | arr[hg19]12p13.33p13.32(173,786-3,543,326) x1dn | 32 | 24+1 | Abnormal NIPT | - | TOP |
|  | arr[hg19]8p23.3p23.1(158,048-7,044,046) x1，arr[hg19]8p23.1p11.22(12,532,885-39,678,723) x3 | 34 | 27+6 | CNS malformations | - | TOP |
|  | arr[hg19] Xp22.33 or Yp11.32(313,342-1,234,634 or 263,342-1,184,634) x1 | 29 | 30+0 | Positive second trimester DS screening result | - | TOP |
|  | arr[hg19]15q13.2q13.3(31,104,220-32,915,723) x3 | 26 | 26+5 | Facial malformations | - | TOP |
|  | arr[hg19]8p23.3p11.21(158,048-41,283,514) x3 | 33 | 26+6 | Multiple malformations | - | TOP |
|  | arr[hg19]5p15.33p15.1(113,576-17,625,620) x1 | 29 | 34+4 | SGA | Normal | TOP |
|  | arr[hg19]3p26.3p26.1(61,891-4,327,836) x1， arr[hg19]7p22.3p21.2(163,002-15,054,804) x3，arr[hg19]11q22.1q25(100,024,019-134,937,416) x3 |  | 24+4 | Abnormal CMA | Normal | TOP |
|  | arr[hg19]22q11.21(18,636,749-21,800,471) x1 | 25 | 24+0 | Cardiovascular malformations | - | TOP |
|  | arr[hg19]4p16.3p15.2(68,345-25,296,039) x3，arr[hg19]7q34q36.3(142,044,268-159,119,707) x1 | 29 | 29+0 | Abnormal NIPT | Normal | TOP |
|  | arr[hg19]7p22.3p12.1(162,702-51,548,268) x3,  arr[hg19] 9p24.3(208,454-823,768) x1 | 29 | 25+0 | Multiple malformations | - | TOP |
|  | arr[hg19]22q11.21(18,648,855-20,312,661) x1 | 36 | 25+2 | Cardiovascular malformations | - | LB |
|  | arr[hg19]13q11q12.12(19,436,286-24,296,868) x3 | 27 | 26+0 | Multiple malformations | - | TOP |
|  | arr[hg19]17q12(34,822,465-36,307,773) x1dn | 37 | 30+1 | Urogenital malformations | - | TOP |
|  | arr[hg19]16p11.2(29,428,531-30,350,748) x1 | 32 | 25+2 | Urogenital malformations | - | LB |
|  | arr[hg19]7q11.23(72,723,370-74,154,209) x1 | 44 | 31+1 | FGR | - | PTB |
|  | arr[hg19]2q37.2q37.3(236,664,895-242,782,258) x1 | 28 | 24+6 | Urogenital malformations | - | TOP |
|  | arr[hg19]2q37.3(239,928,338-242,782,258) x1 | 36 | 26+0 | Thoracic abnormalities | - | TOP |
|  | arr[hg19]Xp22.31(6,455,151-8,135,568) x0 | 31 | 24+0 | Facial malformations | - | TOP |
|  | arr[hg19]22q13.2q13.33(43,449,816-51,197,766) x1 | 27 | 24+4 | Urogenital malformations | - | TOP |
|  | arr[hg19]4p16.3p16.1(796,111-8,721,580) x1 | 29 | 24+6 | FGR | - | TOP |
|  | arr[hg19]22q11.21(18,631,364-21,800,471) x1 | 26 | 25+6 | Skeletal malformations | - | TOP |
| ***Uncertain Result*** | ***63*** |  |  |  |  |  |
| VUS  （51） | arr[hg19]7q14.1(38,842,718-39,352,266)×3 | 26 | 27+0 | Skeletal malformations | 46，X，inv（Y）(p11q11),?(20)(p11.2) | TOP |
|  | arr[hg19]6p12.3q15(49,625,289-91,133,167) hmz | 28 | 25+0 | Facial malformations | - | TOP |
|  | arr[hg19]4p16.3p12(68,345-45,810,772) x3 | 20 | 31+0 | Urogenital malformations | - | TOP |
|  | arr[hg19]Yq11.221q11.222(19,588,384-21,028,944)x0 | 22 | 29+6 | Multiple malformations | - | TOP |
|  | arr[hg19]2p12(78,484,700 - 80,089,819)×4 | 27 | 25+2 | Facial malformations | - | TOP |
|  | arr[hg19]9q21.12q21.33 (72,265,36687,478,135)hmz | 23 | 32+0 | Polyhydramnios | - | TOP |
|  | arr[hg19]1p32.3(54,846,200-55,355,662)x3 | 26 | 34+0 | CNS malformations | - | LB |
|  | LOH | 36 | 30+5 | CNS malformations | - | IUD |
|  | arr[hg19]6p24.3(7,897,062-9,521,761)x3 | 37 | 27+0 | Positive second trimester DS screening result | - | LB |
|  | arr[hg19]Xq28(154,943,961-155,233,098) x3 | 29 | 26+5 | SGA | - | LB |
|  | arr[hg19]13q13.3(37,156,327-38,571,508) x1 | 27 | 26+5 | CNS malformations | - | LB |
|  | arr[hg19]7q11.22(70,236,723-70,705,107) x3 | 23 | 35+5 | Cardiovascular malformations | - | LB |
|  | arr[hg19]5p12p11(45,580,147-46,332,353) x3 | 28 | 25+0 | CNS malformations | Normal | TOP |
|  | arr[hg19]Xq28(152,916,789-153,029,189) x1 | 23 | 31+6 | Multiple malformations | - | TOP |
|  | arr[hg19]16p11.2(29,591,326-30,178,406) x3 | 24 | 25+4 | Cardiovascular malformations | - | LB |
|  | arr[hg19]6q24.1(140,944,532-141,969,994) x3 | 21 | 27+0 | CNS malformations | - | TOP |
|  | arr[hg19]7q31.1(110,868,170-111,340,760) x1 | 35 | 29+2 | Increased NT | - | LB |
|  | arr[hg19]17p13.2(4,007,665-4,555,135) x3 | 23 | 28+6 | Polyhydramnios | Normal | LB |
|  | XX (mos)1.74 | 26 | 32+2 | Urogenital malformations | - | LB |
|  | arr[hg19]14q21.1(38,617,015-39,439,526)x1 | 35 | 33+4 | SGA | - | LB |
|  | arr[hg19]16p12.2(21,405,327-21,931,248) x1 | 28 | 27+6 | Urogenital malformations | Normal | TOP |
|  | arr[hg19]22q11.21q11.22(20,964,245-22,769,923) x3 | 29 | 32+0 | Urogenital malformations | - | TOP |
|  | arr[hg19]1p36.32(3,540,373-4,089,613) x3 | 24 | 29+4 | FGR | - | LB |
|  | arr[hg19]16p12.2(21,405,327-21,931,248) x1 | 28 | 30+2 | Polyhydramnios | - | TOP |
|  | arr[hg19]22q11.22q11.23(22,997,928-25,043,045) x3 | 24 | 25+0 | Abnormal NIPT | - | TOP |
|  | arr[hg19]1p31.1(73,339,931-75,828,445) x3 | 24 | 24+0 | Urogenital malformations | TNNI3K | TOP |
|  | arr[hg19]16p13.11(14,892,975-16,527,659) x1 | 26 | 28+0 (MCDA) | Cardiovascular malformations | Normal | TOP |
|  |  |  |  | Normal | Normal | LB |
|  | arr[hg19]10q23.1(84,221,372-85,307,085) x4 | 38 | 37+0 | SGA | - | LB |
|  | arr[hg19]10q21.1(53,678,343-55,476,600) x1 mat，arr[hg19] 10q11.22q11.23(46,252,072-51,817,663) x3 mat | 22 | 30+5 | SGA | - | LB |
|  | arr[hg19]1q44(245,521,981-246,614,471) x1 mat | 25 | 25+5 | Cardiovascular malformations | - | LB |
|  | arr[hg19]5q21.2q21.3(103,557,086-105,405,016) x3 | 28 | 32+5 | Skeletal malformations | - | TOP |
|  | arr[hg19]13q21.2(60,321,686-60,709,021) x1 pat | 36 | 24+0 | Cardiovascular malformations | - | TOP |
|  | arr[hg19]7q21.2(91,269,594-91,785,021) x1 mat | 30 | 24+3 | Cardiovascular malformations | Normal | TOP |
|  | arr[hg19]15q13.2q13.3(30,386,398-32,444,043) x1 | 36 | 32+2 | Abnormal NIPT | - | TOP |
|  | arr[hg19]6p12.1(53,311,803-56,719,541) x3 mat | 28 | 24+0 | Abnormal NIPT | - | LB |
|  | arr[hg19]9p24.2p24.1(4,045,207-5,287,502) x1 | 26 | 29+5 | Cardiovascular malformations | - | LB |
|  | arr[hg19]1q21.3q23.2(152,250,211-160,474,715) x3 | 31 | 33+3 | Ascites | - | LB |
|  | arr[hg19]2q37.1(232,007,101-234,437,428) x1 | 31 | 24+2 | CNS malformations | - | LB |
|  | LOH 15p |  | 25+5 | Abnormal CMA | - | TOP |
|  | arr[hg19]3p22.1p21.31(41,940,980-46,722,973) x3 | 29 | 38+0 | CNS malformations | - | LB |
|  | arr[hg19]Xp22.31(6,455,151-8,135,568) x3 | 29 | 32+0 | Oligohydramnios | - | LB |
|  | LOH 3 | 29 | 32+0 | Multiple malformations | Normal | IUD |
|  | arr[hg19]22q11.21(21,059,669-21,800,471) x1 | 37 | 24+5 | CNS malformations | - | TOP |
|  | arr[hg19]16p13.11(15,058,820-16,309,046) x3 | 24 | 34+2 | Urogenital malformations | - | LB |
|  | arr[hg19]16p13.11(15,058,820-16,309,046) x3 | 32 | 30+5 | Multiple malformations | - | TOP |
|  | arr[hg19]18q22.3(68,818,406-70,628,255) x1 | 21 | 25+3 | Skeletal malformations | - | TOP |
|  | arr[hg19]12q14.3(66,835,964-67,536,493) x1 | 23 | 25+6 | Urogenital malformations | - | TOP |
|  | arr[hg19]2q21.2q21.3(134,963,256-135,984,068) x1 | 26 | 25+0 | Urogenital malformations | - | TOP |
|  | arr[hg19]5p15.2(12,115,804-13,495,391) x1 | 24 | 29+2 | CNS malformations | - | TOP |
|  | UPD 11 | 24 | 24+0 | Polyhydramnios | - | TOP |
| Likely pathogenic  (7) | arr[hg19]16p11.2(29,351,826-30,190,029)x1 dn | 38 | 24+0 | Abnormal NIPT | - | LB |
|  | arr[hg19]20q11.21q12(29,846,302-38,591,841) x3 dn | 44 | 26+1 | CNS malformations | - | TOP |
|  | arr[hg19]5p13.2(36,746,516-37,410,586) x1 dn | 21 | 26+4 | Urogenital malformations | - | TOP |
|  | arr[hg19]17p13.3(999,008-1,583,647) x3 dn | 29 | 26+0 | Urogenital malformations | - | PTB |
|  | arr[hg19]16p13.11(14,892,975-16,527,659) x1 | 21 | 30+0 | FGR | - | LB |
|  | arr[hg19]16p11.2(29,428,531-30,190,029) x1 dn | 29 | 25+0 | CNS malformations | - | TOP |
|  | arr[hg19]1q31.1q31.3(187,769,077-195,376,998) x1 mat | 25 | 27+0 | Abnormal NIPT | - | LB |
| Likely Benign  (5) | arr[hg19]16p12.2 (21,816,542-22,431,357)x3 pat | 30 | 25+0 | Urogenital malformations | - | LB |
|  | arr[hg19]8q22.2(99,558,638-100,578,926)x3 mat | 32 | 28+0 | Urogenital malformations | - | LB |
|  | arr[hg19]3p12.3(76,329,290-79,621,422) x3 mat | 28 | 27+0 | CNS malformations | - | LB |
|  | arr[hg19]2q13(111,382,573-113,111,856) x1 pat | 27 | 29+1 | CNS malformations | - | PTB |
|  | arr[hg19]16q23.2(80,335,571-81,660,608) x3 mat | 28 | 30+0 | CNS malformations | - | LB |
| Total | 204 |  |  |  |  |  |

Abbreviations: CNV-Copy number variants; PTB-Preterm birth; IUD-Intrauterine death; SGA-Small for gestational age; FGR-Fetal growth restriction; NIPT-Noninvasive prenatal testing; FISH- Fluorescence in situ hybridization; CMA-Chromosomal microarray analysis; CNS malformations-Central nervous system malformations; VUS-Variants of uncertain significance; LB-Live birth; TOP-Termination of the pregnancy.

**Table S3 Summary of the complications of routine amniocentesis**

| Complication | Amniocentesis to Complication interval | Gestation | Age | Timing of amniocentesis | Indication of  amniocentesis | Genomic  result | | Genomic results  before delivery  (Y/N) | Fetal  outcome |
| --- | --- | --- | --- | --- | --- | --- | --- | --- | --- |
| **PTB**  **(39)** | Within one week  (2) | Singleton (2) | 37 | 22+3 | Advanced maternal age | | Normal | N | LB |
|  |  |  | 30 | 23+4 | Cardiovascular malformations | | Normal | N | LB |
|  | Within one month  (6) | Singleton (3) | 27 | 16+0 | Positive second trimester DS screening result | | CNV | Y | LB |
|  |  |  | 30 | 23+2 | FGR | | Normal | Y | LB |
|  |  |  | 38 | 22+1 | Skeletal malformations | | Normal | Y | LB |
|  |  | MCDA (2 ) | 29 | 22+4 | Cardiovascular malformations | | Normal | Y | LB |
|  |  |  |  |  | CNS malformations | | Normal | Y | LB |
|  |  |  | 25 | 21+0 | Cardiovascular malformations | | Normal | Y | LB |
|  |  |  |  |  | Patient’s request | | Normal | Y | LB |
|  |  | DCDA (1 ) | 27 | 16+5 | Patient’s request | | Normal | Y | LB |
|  |  |  |  |  | Increased NT | | Normal | Y | LB |
|  | After one month  (31) | Singleton (17) | 27 | 19+0 | Multiple malformations | | Normal | Y | LB |
|  |  |  | 40 | 16+3 | Advanced maternal age | | Normal | Y | LB |
|  |  |  | 39 | 17+0 | Positive second trimester DS screening result | | Normal | Y | LB |
|  |  |  | 30 | 23+4 | Cardiovascular malformations | | Normal | Y | LB |
|  |  |  | 38 | 22+1 | Skeletal malformations | | Normal | Y | LB |
|  |  |  | 39 | 17+0 | Abnormal NIPT | | Normal | Y | LB |
|  |  |  | 33 | 21+5 | Abnormal NIPT | | VUS | Y | LB |
|  |  |  | 36 | 18+0 | Abnormal tribunal history | | Normal | Y | LB |
|  |  |  | 32 | 22+5 | Cardiovascular malformations | | Normal | Y | LB |
|  |  |  | 39 | 17+0 | Abnormal tribunal history | | Normal | Y | LB |
|  |  |  | 32 | 17+0 | Family history of genetic disease | | Normal | Y | LB |
|  |  |  | 25 | 17+5 | Family history of genetic disease | | VUS | Y | LB |
|  |  |  | 28 | 17+1 | Family history of genetic disease | | Normal | Y | LB |
|  |  |  | 30 | 22+0 | Skeletal malformations | | Normal | Y | LB |
|  |  |  | 29 | 22+0 | Increased NT | | Normal | Y | LB |
|  |  |  | 40 | 17+6 | Increased NT | | Normal | Y | LB |
|  |  |  | 30 | 16+0 | Family history of genetic disease | | Normal | Y | LB |
|  |  | MCDA  (5) | 40 | 20+5 | CNS malformations | | Normal | Y | LB |
|  |  |  |  |  | Patient’s request | | Normal | Y | LB |
|  |  |  | 27 | 21+0 | Urogenital malformations | | Normal | Y | LB |
|  |  |  |  |  | Multiple malformations | | Normal | Y | LB |
|  |  |  | 36 | 17+0 | Increased NT | | Normal | Y | LB |
|  |  |  |  |  | Patient’s request | | Normal | Y | LB |
|  |  |  | 30 | 18+2 | Increased NT | | Normal | Y | LB |
|  |  |  |  |  | Patient’s request | | Normal | Y | LB |
|  |  |  | 29 | 16+3 | Increased NT | | Normal | Y | LB |
|  |  |  |  |  | Patient’s request | | Normal | Y | LB |
|  |  | DCDA  (9) | 36 | 16+0 | Increased NT | | Normal | Y | LB |
|  |  |  |  |  | Patient’s request | | Normal | Y | LB |
|  |  |  | 34 | 17+0 | Increased NT | | Normal | Y | LB |
|  |  |  |  |  | Patient’s request | | Normal | Y | LB |
|  |  |  | 37 | 18+0 | CNS malformations | | Normal | Y | LB |
|  |  |  |  |  | CNS malformations | | Normal | Y | LB |
|  |  |  | 40 | 20+0 | Abnormal NIPT | | T18 | Y | LB |
|  |  |  |  |  | Abnormal NIPT | | Normal | Y | LB |
|  |  |  | 33 | 19+2 | Increased NT | | Normal | Y | LB |
|  |  |  |  |  | Patient’s request | | Normal | Y | LB |
|  |  |  | 25 | 19+4 | Increased NT | | T21 | Y | LB |
|  |  |  |  |  | Patient’s request | | Normal | Y | LB |
|  |  |  | 30 | 17+0 | Abnormal NIPT | | Normal | Y | LB |
|  |  |  |  |  | Abnormal NIPT | | Normal | Y | LB |
|  |  |  | 35 | 16+3 | Abnormal NIPT | | T21 | Y | LB |
|  |  |  |  |  | Abnormal NIPT | | Normal | Y | LB |
|  |  |  | 33 | 17+6 | Abnormal NIPT | | Normal | Y | LB |
|  |  |  |  |  | Abnormal NIPT | | Normal | Y | LB |
| **IUD**  **(49)** | Within one week  (12) | Singleton (6) | 26 | 23+2 | Multiple malformations | | Normal | N | IUD |
|  |  |  | 32 | 18+5 | Skeletal malformations | | Normal | N | IUD |
|  |  |  | 33 | 17+5 | Abnormal NIPT | | Normal | N | IUD |
|  |  |  | 34 | 16+0 | Abnormal NIPT | | Normal | N | IUD |
|  |  |  | 25 | 19+2 | Ascites | | Normal | N | IUD |
|  |  |  | 32 | 17+0 | Family history of genetic disease | | Normal | N | IUD |
|  |  | MCDA  (1) | 31 | 18+0 | Multiple malformations | | Normal | N | IUD |
|  |  |  |  |  | Multiple malformations | | Normal | N | IUD |
|  |  | DCDA  (2) | 35 | 24+0 | CNS malformations | | Normal | N | IUD |
|  |  |  |  |  | Patient’s request | | Normal | N | IUD |
|  |  |  | 27 | 16+0 | Abnormal NIPT | | T21 | N | IUD |
|  |  |  |  |  | Abnormal NIPT | | Normal | N | IUD |
|  | Within one month  (28) | Singleton  (6) | 28 | 22+0 | Chest malformations | | Normal | Y | IUD |
|  |  |  | 40 | 18+0 | Abnormal NIPT | | Normal | Y | IUD |
|  |  |  | 28 | 18+5 | Positive second trimester DS screening result | | Normal | Y | IUD |
|  |  |  | 37 | 17+3 | Abnormal NIPT | | Normal | Y | IUD |
|  |  |  | 35 | 16+0 | Abnormal karyotyping result | | Normal | Y | IUD |
|  |  |  | 31 | 16+0 | Increased NT | | Normal | Y | IUD |
|  |  | MCDA（5） | 28 | 22+0 | Patient’s request | | Normal | Y | IUD |
|  |  |  |  |  | Patient’s request | | Normal | Y | IUD |
|  |  |  | 35 | 18+0 | Abnormal NIPT | | Normal | Y | IUD |
|  |  |  |  |  | Abnormal NIPT | | Normal | Y | IUD |
|  |  |  | 29 | 20+1 | CNS malformations | | Normal | Y | IUD |
|  |  |  |  |  | Patient’s request | | Normal | Y | IUD |
|  |  |  | 31 | 20+6 | Cardiovascular malformations | | Normal | Y | IUD |
|  |  |  |  |  | Patient’s request | | Normal | Y | IUD |
|  |  |  | 36 | 16+0 | Abnormal NIPT | | T21 | Y | IUD |
|  |  |  |  |  | Abnormal NIPT | | Normal | Y | IUD |
|  |  | DCDA（6） | 28 | 22+0 | Urogenital malformations | | Normal | Y | IUD |
|  |  |  |  |  | Patient’s request | | Normal | Y | IUD |
|  |  |  | 33 | 23+0 | Cardiovascular malformations | | Normal | Y | IUD |
|  |  |  |  |  | Cardiovascular malformations | | Normal | Y | IUD |
|  |  |  | 34 | 23+0 | Multiple malformations | | Normal | Y | IUD |
|  |  |  |  |  | Patient’s request | | Normal | Y | IUD |
|  |  |  | 27 | 16+4 | Abnormal NIPT | | Normal | Y | IUD |
|  |  |  |  |  | Patient’s request | | Normal | Y | IUD |
|  |  |  | 33 | 19+0 | Digestive malformations | | T21 | Y | IUD |
|  |  |  |  |  | CNS malformations | | Normal | Y | IUD |
|  |  |  | 34 | 16+0 | Abnormal tribunal history | | Normal | Y | IUD |
|  |  |  |  |  | Abnormal tribunal history | | Normal | Y | IUD |
|  | After one month  (9) | Singleton (5) | 31 | 19+5 | CNS malformations | | Normal | Y | IUD |
|  |  |  | 27 | 21+4 | Abnormal karyotyping result | | T21 | Y | IUD |
|  |  |  | 25 | 20+0 | Abnormal NIPT | | T21 | Y | IUD |
|  |  |  | 37 | 17+3 | Abnormal NIPT | | Normal | Y | IUD |
|  |  |  | 21 | 16+2 | Increased NT | | VUS | Y | IUD |
|  |  | MCDA  (1) | 30 | 18+0 | Increased NT | | Normal | Y | IUD |
|  |  |  |  |  | Increased NT | | Normal | Y | IUD |
|  |  | DCDA  (1) | 34 | 16+0 | Abnormal CMA | | Normal | Y | IUD |
|  |  |  |  |  | Abnormal CMA | | Normal | Y | IUD |
| Total | 88 |  |  |  |  | |  |  |  |

Abbreviations: PTB-Preterm birth; IUD-Intra uterine death; MCDA-Monochorionic diamniotic pregnancies with double puncture; DCDA-Dichorionic diamniotic twin pregnancies with double puncture; NT-Nuchal translucency; FGR-Fetal growth restriction; NIPT-Noninvasive prenatal testing; CMA-Chromosomal microarray analysis; CNS malformations-Central nervous system malformations; VUS-Variants of uncertain significance; LB-Live birth; TOP-Termination of the pregnancy.

**Table S4 CMA results of routine amniocentesis.**

| CMA results | Details | Maternal  Age | Timing of amniocentesis | Indication of  amniocentesis | Karyotyping  (-/results) | Fetal  outcome |
| --- | --- | --- | --- | --- | --- | --- |
| ***pathogenic*** | ***335*** |  |  |  |  |  |
| Aneuploidies  (261) | Trisomy 21（132） | 32 | 21+1 | Cardiovascular malformations | Normal | TOP |
|  |  | 21 | 16+0 | Increased NT | - | TOP |
|  |  | 29 | 13+0 | Increased NT | - | TOP |
|  |  | 39 | 21+0 | Advanced maternal age | - | LB |
|  |  | 22 | 18+6 | Increased NT | - | TOP |
|  |  | 28 | 16+0 | Positive second trimester DS screening result | - | LB |
|  |  | 32 | 19+5 | Multiple malformations | - | TOP |
|  |  | 24 | 20+3 | Abnormal NIPT | - | TOP |
|  |  | 31 | 20+6 | Increased NT | - | TOP |
|  |  | 39 | 22+5 | Increased NT | - | TOP |
|  |  | 40 | 17+0 | Positive second trimester DS screening result | - | TOP |
|  |  | 28 | 19+0 | Multiple malformations | - | TOP |
|  |  | 26 | 16+1 | Abnormal NIPT | - | TOP |
|  |  | 43 | 16+0 | Abnormal NIPT | - | TOP |
|  |  | 32 | 23+0 | Cardiovascular malformations | - | TOP |
|  |  | 42 | 17+0 | Abnormal NIPT | - | TOP |
|  |  | 32 | 18+2 | Increased NT | - | TOP |
|  |  | 44 | 18+0 | Positive second trimester DS screening result | - | TOP |
|  |  | 28 | 22+4 | Abnormal NIPT | - | TOP |
|  |  | 27 | 21+4 | Abnormal karyotyping result | Normal | IUD |
|  |  | 29 | 16+3 | Fetal tumor | - | TOP |
|  |  | 30 | 19+0 | Cardiovascular malformations | - | TOP |
|  |  | 42 | 19+6 | Abnormal NIPT | - | TOP |
|  |  | 30 | 23+0 | Abnormal NIPT | - | TOP |
|  |  | 32 | 18+0 | Increased NT | - | TOP |
|  |  | 39 | 20+6 | Advanced maternal age | - | TOP |
|  |  | 34 | 23+4 | Multiple malformations | - | TOP |
|  |  | 18 | 22+0 | Abnormal NIPT | - | TOP |
|  |  | 24 | 21+2 | Multiple malformations | - | TOP |
|  |  | 39 | 19+0 | Increased NT | - | TOP |
|  |  | 29 | 16+3 | Abnormal NIPT | - | TOP |
|  |  | 40 | 16+0 | Chest malformations | - | LB |
|  |  | 44 | 18+0 | Abnormal NIPT | - | TOP |
|  |  | 19 | 17+0 | Increased NT | - | TOP |
|  |  | 30 | 17+3 | Abnormal NIPT | - | TOP |
|  |  | 35 | 18+0 | Advanced maternal age | - | TOP |
|  |  | 23 | 18+0 | Increased NT | - | TOP |
|  |  | 38 | 17+3 | Abnormal NIPT | - | TOP |
|  |  | 43 | 16+0 | Abnormal NIPT | - | TOP |
|  |  | 33 | 17+0 | Cardiovascular malformations | - | TOP |
|  |  | 31 | 18+4 | Abnormal NIPT | - | TOP |
|  |  | 37 | 20+3 | Advanced maternal age | - | LB |
|  |  | 27 | 23+0 | CNS malformations | - | TOP |
|  |  | 29 | 16+0 | Abnormal NIPT | - | TOP |
|  |  | 40 | 17+0 | Abnormal NIPT | - | TOP |
|  |  | 38 | 17+1 | Abnormal NIPT | - | TOP |
|  |  | 25 | 18+2 | Abnormal NIPT | - | TOP |
|  |  | 27 | 22+2 | Abnormal NIPT | - | TOP |
|  |  | 27 | 19+5 | Abnormal NIPT | - | TOP |
|  |  | 33 | 22+0 | Abnormal NIPT | - | TOP |
|  |  | 34 | 21+0 | Abnormal NIPT | - | TOP |
|  |  | 37 | 21+3 | CNS malformations | - | TOP |
|  |  | 28 | 17+0 | Abnormal NIPT | - | TOP |
|  |  | 35 | 16+0 | Fetal tumor | - | TOP |
|  |  | 38 | 23+0 | Cardiovascular malformations | - | TOP |
|  |  | 29 | 17+0 | Multiple malformations | - | TOP |
|  |  | 33 | 23+0 | Abnormal NIPT | - | TOP |
|  |  | 44 | 21+0 | Positive second trimester DS screening result | - | TOP |
|  |  | 27 | 20+0 | Abnormal NIPT | - | TOP |
|  |  | 31 | 20+0 | Abnormal NIPT | - | TOP |
|  |  | 43 | 16+0 | Increased NT | - | TOP |
|  |  | 39 | 19+0 | Abnormal NIPT | - | TOP |
|  |  | 25 | 19+0 | Abnormal NIPT | - | TOP |
|  |  | 37 | 18+0 | Abnormal NIPT | - | TOP |
|  |  | 27 | 16+0 | Abnormal NIPT | - | IUD |
|  |  | 32 | 19+0 | Abnormal NIPT | - | TOP |
|  |  | 35 | 16+0 | Abnormal NIPT | - | TOP |
|  |  | 43 | 16+2 | Increased NT | - | TOP |
|  |  | 38 | 16+0 | Abnormal NIPT | - | TOP |
|  |  | 35 | 18+0 | Abnormal NIPT | - | TOP |
|  |  | 36 | 18+2 | Abnormal NIPT | - | TOP |
|  |  | 43 | 18+0 | Abnormal NIPT | - | TOP |
|  |  | 43 | 18+0 | Abnormal NIPT | - | TOP |
|  |  | 35 | 17+3 | Abnormal NIPT | - | LB |
|  |  | 31 | 20+5 | Abnormal NIPT | - | TOP |
|  |  | 33 | 18+2 | Abnormal NIPT | - | TOP |
|  |  | 34 | 22+5 | Abnormal NIPT | - | TOP |
|  |  | 39 | 16+2 | Abnormal NIPT | - | TOP |
|  |  | 41 | 17+5 | Abnormal NIPT | - | TOP |
|  |  | 37 | 16+2 | Abnormal NIPT | - | TOP |
|  |  | 37 | 16+4 | Abnormal NIPT | - | TOP |
|  |  | 29 | 17+5 | Abnormal NIPT | - | TOP |
|  |  | 41 | 20+0 | Abnormal NIPT | - | TOP |
|  |  | 32 | 16+1 | Abnormal NIPT | - | TOP |
|  |  | 32 | 16+0 | Abnormal NIPT | - | TOP |
|  |  | 26 | 18+0 | Abnormal NIPT | - | LB |
|  |  | 32 | 18+0 | Increased NT | - | TOP |
|  |  | 33 | 18+0 | Abnormal NIPT | - | TOP |
|  |  | 21 | 17+0 | Increased NT | - | TOP |
|  |  | 38 | 16+0 | Abnormal NIPT | - | TOP |
|  |  | 39 | 16+0 | Abnormal NIPT | - | TOP |
|  |  | 23 | 23+0 | Abnormal NIPT | - | TOP |
|  |  | 29 | 16+6 | Abnormal NIPT | - | TOP |
|  |  | 26 | 20+0 | Abnormal NIPT | - | TOP |
|  |  | 31 | 18+0 | Positive second trimester DS screening result | - | TOP |
|  |  | 31 | 18+6 | Abnormal NIPT | - | TOP |
|  |  | 31 | 18+6 | Abnormal NIPT | - | TOP |
|  |  | 39 | 16+1 | Increased NT | - | LB |
|  |  | 33 | 19+0 | Digestive malformations | - | IUD |
|  |  | 25 | 18+1 | CNS malformations | - | TOP |
|  |  | 34 | 16+5 | Abnormal NIPT | - | TOP |
|  |  | 32 | 18+0 | Abnormal NIPT | - | TOP |
|  |  | 28 | 18+1 | Abnormal karyotyping result | - | TOP |
|  |  | 46 | 16+5 | Increased NT | - | TOP |
|  |  | 42 | 20+2 | Abnormal NIPT | - | TOP |
|  |  | 34 | 21+0 | Abnormal NIPT | - | TOP |
|  |  | 37 | 16+0 | Increased NT | - | TOP |
|  |  | 35 | 18+4 | Abnormal NIPT | - | TOP |
|  |  | 31 | 22+0 | Abnormal NIPT | - | TOP |
|  |  | 32 | 19+0 | Increased NT | - | TOP |
|  |  | 29 | 19+5 | Abnormal NIPT | - | TOP |
|  |  | 36 | 16+5 | Abnormal NIPT | - | TOP |
|  |  | 41 | 21+0 | Abnormal NIPT | - | TOP |
|  |  | 26 | 17+2 | Abnormal NIPT | - | TOP |
|  |  | 25 | 22+4 | Abnormal NIPT | - | TOP |
|  |  | 24 | 19+0 | Abnormal NIPT | - | TOP |
|  |  | 32 | 20+0 | Abnormal NIPT | - | TOP |
|  |  | 27 | 20+3 | Increased NT | - | TOP |
|  |  | 30 | 19+0 | Abnormal NIPT | - | TOP |
|  |  | 31 | 19+0 | Abnormal NIPT | - | TOP |
|  |  | 25 | 19+4 | Increased NT | - | PTB |
|  |  | 36 | 16+6 | Abnormal NIPT | - | TOP |
|  |  | 28 | 19+0 | Abnormal NIPT | - | TOP |
|  |  | 37 | 16+5 | Abnormal NIPT | - | LB |
|  |  | 25 | 17+6 | Abnormal NIPT | - | TOP |
|  |  | 45 | 16+0 | Abnormal NIPT | - | TOP |
|  |  | 27 | 21+3 | Abnormal NIPT | - | TOP |
|  |  | 40 | 18+4 | Abnormal NIPT | - | TOP |
|  |  | 42 | 17+3 | Advanced maternal age | - | TOP |
|  |  | 33 | 17+2 | Increased NT | - | TOP |
|  |  | 39 | 23+4 | Abnormal NIPT | - | TOP |
|  |  | 43 | 16+0 | Abnormal NIPT | - | TOP |
|  | Trisomy 18（35） | 29 | 19+0 | Multiple malformations | - | TOP |
|  |  | 29 | 21+0 | Multiple malformations | - | TOP |
|  |  | 27 | 17+1 | Increased NT | - | TOP |
|  |  | 27 | 17+1 | Patient’s request | - | TOP |
|  |  | 28 | 22+0 | Multiple malformations | - | TOP |
|  |  | 29 | 22+0 | Abnormal NIPT | - | TOP |
|  |  | 34 | 19+0 | Multiple malformations | - | TOP |
|  |  | 42 | 16+6 | Abnormal NIPT | - | TOP |
|  |  | 44 | 16+4 | Abnormal NIPT | - | TOP |
|  |  | 40 | 18+0 | Increased NT | - | TOP |
|  |  | 30 | 17+0 | Abnormal NIPT | - | TOP |
|  |  | 25 | 22+0 | Multiple malformations | - | TOP |
|  |  | 29 | 20+0 | Patient’s request | - | TOP |
|  |  | 41 | 18+4 | Abnormal NIPT | - | TOP |
|  |  | 42 | 20+1 | Abnormal NIPT | - | LB |
|  |  | 41 | 20+0 | Cardiovascular malformations | - | TOP |
|  |  | 33 | 16+0 | Increased NT | - | LB |
|  |  | 38 | 21+5 | Advanced maternal age | - | TOP |
|  |  | 25 | 23+0 | Abnormal NIPT | - | TOP |
|  |  | 29 | 17+0 | Positive second trimester DS screening result | - | TOP |
|  |  | 39 | 24+0 | Abnormal NIPT | - | TOP |
|  |  | 35 | 22+0 | Increased NT | - | TOP |
|  |  | 34 | 16+0 | Abnormal NIPT | - | TOP |
|  |  | 41 | 23+6 | Cardiovascular malformations | - | TOP |
|  |  | 39 | 18+2 | Skeletal malformations | - | TOP |
|  |  | 40 | 20+4 | Multiple malformations | - | TOP |
|  |  | 35 | 18+6 | Increased NT | - | TOP |
|  |  | 24 | 20+4 | Abnormal NIPT | - | TOP |
|  |  | 42 | 18+1 | Abnormal NIPT | - | TOP |
|  |  | 34 | 20+4 | Abnormal NIPT | - | TOP |
|  |  | 27 | 16+0 | Abnormal NIPT | - | TOP |
|  |  | 40 | 20+0 | Abnormal NIPT | - | PTB |
|  |  | 42 | 18+0 | Multiple malformations | - | TOP |
|  |  | 26 | 16+0 | Abnormal NIPT | - | TOP |
|  |  | 35 | 17+0 | Multiple malformations | - | TOP |
|  | Trisomy 13（19） | 29 | 22+0 | CNS malformations | - | TOP |
|  |  | 32 | 19+3 | Positive second trimester DS screening result | - | LB |
|  |  | 32 | 20+4 | Facial malformations | - | TOP |
|  |  | 22 | 23+5 | Multiple malformations | - | TOP |
|  |  | 33 | 22+0 | Multiple malformations | - | TOP |
|  |  | 26 | 16+0 | Multiple malformations | - | TOP |
|  |  | 42 | 21+0 | Abnormal NIPT | - | TOP |
|  |  | 40 | 16+1 | Increased NT | - | TOP |
|  |  | 42 | 22+5 | Advanced maternal age | - | TOP |
|  |  | 29 | 18+1 | CNS malformations | - | TOP |
|  |  | 40 | 17+2 | Increased NT | - | TOP |
|  |  | 26 | 18+1 | Multiple malformations | - | TOP |
|  |  | 26 | 17+0 | Multiple malformations | - | TOP |
|  |  | 23 | 17+2 | Increased NT | - | TOP |
|  |  | 23 | 17+0 | Increased NT | - | TOP |
|  |  | 28 | 16+0 | Abnormal NIPT | - | TOP |
|  |  | 30 | 23+0 | Multiple malformations | - | TOP |
|  |  | 28 | 23+0 | Multiple malformations | - | TOP |
|  |  | 42 | 18+2 | Cardiovascular malformations | - | TOP |
|  | Trisomy 2 | 27 | 18+2 | Abnormal NIPT | - | TOP |
|  |  | 28 | 18+1 | Abnormal NIPT | - | TOP |
|  | Trisomy 8 | 37 | 17+0 | Abnormal NIPT | - | TOP |
|  | Trisomy 9 | 41 | 17+0 | Abnormal NIPT | - | TOP |
|  | Trisomy 10 | 41 | 16+0 | Abnormal NIPT | - | TOP |
|  | Trisomy 16 | 29 | 16+0 | Abnormal NIPT | - | LB |
|  | XYY (13) | 40 | 19+0 | Abnormal NIPT | - | TOP |
|  |  | 36 | 16+0 | Abnormal NIPT | - | TOP |
|  |  | 34 | 21+1 | Abnormal NIPT | - | TOP |
|  |  | 28 | 19+4 | Abnormal NIPT | - | TOP |
|  |  | 32 | 19+0 | Abnormal NIPT | - | TOP |
|  |  | 36 | 16+6 | Abnormal NIPT | - | TOP |
|  |  | 29 | 19+0 | Abnormal NIPT | - | TOP |
|  |  | 33 | 18+4 | Abnormal NIPT | - | TOP |
|  |  | 28 | 16+0 | Abnormal NIPT | - | TOP |
|  |  | 36 | 16+3 | Abnormal NIPT | - | LB |
|  |  | 26 | 19+0 | Abnormal NIPT | - | TOP |
|  |  | 39 | 17+0 | Abnormal NIPT | - | TOP |
|  |  | 30 | 19+0 | Abnormal NIPT | - | LB |
|  | XO（18） | 31 | 18+0 | Multiple malformations | - | TOP |
|  |  | 29 | 14+0 | Multiple malformations | - | TOP |
|  |  | 28 | 20+0 | Multiple malformations | - | TOP |
|  |  | 39 | 16+0 | Fetal tumor | - | TOP |
|  |  | 26 | 20+1 | Multiple malformations | - | TOP |
|  |  | 35 | 21+1 | Abnormal NIPT | XO | TOP |
|  |  | 24 | 17+6 | Abnormal CMA result | - | TOP |
|  |  | 39 | 16+5 | Advanced maternal age | - | LB |
|  |  | 37 | 23+0 | Abnormal NIPT | - | TOP |
|  |  | 34 | 19+1 | Abnormal NIPT | - | LB |
|  |  | 24 | 19+5 | Multiple malformations | - | TOP |
|  |  | 38 | 24+0 | Abnormal CMA result | - | TOP |
|  |  | 32 | 19+1 | Multiple malformations | - | TOP |
|  |  | 29 | 17+0 | Multiple malformations | - | TOP |
|  |  | 37 | 17+0 | Abnormal NIPT | - | TOP |
|  |  | 30 | 20+0 | Abnormal NIPT | - | TOP |
|  |  | 26 | 19+0 | Abnormal NIPT | - | TOP |
|  |  | 26 | 16+0 | Abnormal NIPT | - | TOP |
|  | XXX（12） | 21 | 18+0 | Abnormal NIPT | - | LB |
|  |  | 37 | 21+4 | Abnormal NIPT | - | TOP |
|  |  | 40 | 18+3 | Abnormal NIPT | - | TOP |
|  |  | 32 | 18+0 | Abnormal NIPT | - | LB |
|  |  | 30 | 24+0 | Abnormal NIPT | - | TOP |
|  |  | 40 | 19+0 | Urogenital malformations | - | TOP |
|  |  | 40 | 20+3 | Abnormal NIPT | - | LB |
|  |  | 28 | 16+0 | Abnormal NIPT | - | Lost |
|  |  | 34 | 16+1 | Abnormal NIPT | - | TOP |
|  |  | 27 | 17+6 | Abnormal NIPT | - | LB |
|  |  | 39 | 16+1 | Abnormal NIPT | - | LB |
|  |  | 42 | 17+2 | Abnormal NIPT | - | LB |
|  | XXY (26) | 26 | 20+6 | Facial malformations | - | TOP |
|  |  | 38 | 18+6 | Abnormal NIPT | - | TOP |
|  |  | 29 | 18+0 | Abnormal NIPT | - | TOP |
|  |  | 43 | 16+0 | Abnormal NIPT | - | TOP |
|  |  | 26 | 22+0 | Abnormal NIPT | - | TOP |
|  |  | 36 | 22+0 | Abnormal karyotyping result | XXY | TOP |
|  |  | 30 | 22+0 | Abnormal NIPT | - | TOP |
|  |  | 32 | 16+3 | Increased NT | - | TOP |
|  |  | 36 | 16+0 | Abnormal NIPT | - | LB |
|  |  | 43 | 17+0 | Abnormal NIPT | - | TOP |
|  |  | 40 | 22+5 | Abnormal NIPT | - | TOP |
|  |  | 39 | 16+0 | Abnormal NIPT | - | TOP |
|  |  | 30 | 18+0 | Abnormal NIPT | - | TOP |
|  |  | 36 | 16+6 | Abnormal NIPT | - | LB |
|  |  | 41 | 16+4 | Abnormal NIPT | - | TOP |
|  |  | 39 | 17+4 | Abnormal NIPT | - | TOP |
|  |  | 37 | 19+1 | Abnormal NIPT | - | TOP |
|  |  | 26 | 21+0 | Abnormal NIPT | - | TOP |
|  |  | 40 | 16+5 | Abnormal NIPT | - | TOP |
|  |  | 27 | 17+0 | Abnormal NIPT | - | LB |
|  |  | 40 | 18+0 | Abnormal NIPT | - | TOP |
|  |  | 40 | 18+0 | Abnormal NIPT | - | TOP |
|  |  | 36 | 16+0 | Abnormal NIPT | - | TOP |
|  |  | 30 | 18+0 | Abnormal NIPT | - | TOP |
|  |  | 45 | 22+5 | Abnormal NIPT | - | TOP |
|  |  | 21 | 16+4 | Abnormal NIPT | - | TOP |
| CNV  (74) | arr[hg19]7q36.1q36.3(150,915,999-157,332,012) ×1 dn | 29 | 22+2 | Skeletal malformations | - | TOP |
|  | arr[hg19]Xp22.31(6,958,952-7,598,065)x0 | 30 | 19+0 | Abnormal tribunal history | Normal | LB |
|  | arr[hg19]13q32.2q34(99,048,295-115,107,733)x1 | 26 | 16+0 | CNS malformations | - | TOP |
|  | arr[hg19]20q13.2q13.33(52,466,963-62,913,645)x3 | 26 | 21+0 | Urogenital malformations | - | TOP |
|  | arr[hg19]10p15.3p15.1(100,047-4,548,375)x1 | 29 | 20+1 | Patient’s request | - | LB |
|  | arr[hg19]1q43q44 (239,161,487-249,224,684)×1 | 33 | 22+2 | CNS malformations | - | TOP |
|  | arr[hg19]6 q22.1q23.2（116,215,123-134,987,685）x1 | 28 | 16+0 | Multiple malformations | - | TOP |
|  | arr[hg19]22q11.21 (18,648,855-21,800,471)x1 | 29 | 22+2 | Multiple malformations | - | TOP |
|  | arr[hg19]9p24.3p13.1(208,454-38,772,005)x3，arr[hg19]18q23(73,698,166-78,013,728)x1，arr[hg19]Yq11.221q11.23(16,966,152-28,799,654)x0 arr[hg19]18q23(73,698,166-78,013,728)x1，arr[hg19]Yq11.221q11.23(16,966,152-28,799,654)x0 | 26 | 20+4 | Multiple malformations | - | TOP |
|  | arr[hg19] 17q12(34,822,465-36,410,533)x1 | 29 | 20+0 | Abnormal tribunal history | - | Lost |
|  | arr[hg19]Yp11.31q11.221(2,650,424-18,292,329)x2，arr[hg19] Yq11.221q11.23(18,481,644-28,799,654)x0 | 33 | 20+3 | Abnormal CMA result | 45,XO[40]/46,XY[60] | TOP |
|  | arr[hg19]22q11.21(18,916,842-21,800,797)x1 | 24 | 23+4 | Cardiovascular malformations | - | TOP |
|  | arr[hg19]9p24.3p23(203,861-10,402,130)x3；arr[hg19]9p23p21.2(10,407,804-27,847,113)x3 | 26 | 17+4 | Family history of genetic disease | - | Lost |
|  | arr[hg19]Xp22.33p11.1(168,551-58,455,353)x1；Xq13.1q27.3(71,078,650-145,867,912)x1-2；arr[hg19] Yq11.223(24,216,828-24,985,599)x0 | 32 | 21+2 | Abnormal CMA result | 46,X,i(X)(q10)[76]/45, X[24] | TOP |
|  | arr[hg19]2q33.1q37.3(201,375,732-242,782,258)x3 | 32 | 21+0 | Cardiovascular malformations | - | TOP |
|  | arr[hg19]4q32.3q35.2(169,149,847-190,957,460)x1 | 29 | 23+0 | Cardiovascular malformations | - | TOP |
|  | arr[hg19]14q23.3q24.1(65,767,305-68,437,276)x3 | 31 | 18+1 | Fetal tumor | - | TOP |
|  | arr[hg19]9p24.3p24.2(208,454-2,920,085)x1；arr[hg19]11q23.3q25(117,097,362-134,937,416)x3 | 23 | 21+3 | Abnormal CMA result | - | TOP |
|  | arr[hg19]12q24.33(131,994,147-133,777,562)x3；arr[hg19]13q31.3q34(92,191,795-115,107,733)x1 | 31 | 19+1 | Increased NT | - | TOP |
|  | arr[hg19]18p11.32p11.31(136,227-3,644,083)x1；arr[hg19]18q12.3q23(38,442,157-78,013,728)x3 | 25 | 23+0 | Polyhydramnios | - | TOP |
|  | arr[hg19]2q37.3(238,101,561-242,783,384)x1； arr[hg19] 17p13.3p13.2(525-3,656,587)x3； | 31 | 17+0 | Abnormal tribunal history | - | TOP |
|  | arr[hg19]5p13.2q11.1(36,661,774-49,441,945)x3 | 25 | 17+6 | Increased NT | - | TOP |
|  | del Xp,del Xq, MOS del | 30 | 17+0 | Increased NT | - | TOP |
|  | Chr7 LOH | 38 | 23+3 | Abnormal NIPT result | - | TOP |
|  | arr[hg19]15q23q26.3(69,906,546-102,429,040)x3；arr[hg19]Xq24q28(119,173,583-155,233,098)x1 | 35 | 17+6 | Multiple malformations | - | TOP |
|  | arr[hg19]9q34.3(138,613,582-141,018,648)x1；arr[hg19]17q25.1q25.3(74,476,927-81,041,823)x3 | 31 | 17+4 | Increased NT | - | TOP |
|  | arr[hg19]Xp21.1(31,620,870-31,959,958)x3 | 26 | 20+1 | Family history of genetic disease | - | TOP |
|  | arr[hg19]16p13.3p12.1(85,880-27,309,438)x3 | 30 | 18+2 | Cardiovascular malformations | - | TOP |
|  | arr[hg19]4q31.21q35.2(144,830,778-190,957,460)x3；arr[hg19]15q26.2q26.3(97,174,738-102,429,040)x1 | 26 | 20+0 | Increased NT | - | TOP |
|  | arr[hg19] 4p16.3(68,345-4,277,002)x1 dn | 31 | 18+5 | Abnormal NIPT result | - | LB |
|  | arr[hg19] 7p14.1(40,748,621-42,108,287)x1 | 26 | 24+0 | Cardiovascular malformations | - | TOP |
|  | arr[hg19]8q24.12q24.23(121,105,370-136,634,655)x1 | 24 | 19+0 | Increased NT | - | TOP |
|  | del Xp | 26 | 16+6 | Abnormal NIPT | - | TOP |
|  | arr[hg19]10q26.13q26.3(124,383,274-131,949,030)x3；arr[hg19]10q26.3(131,950,631-135,426,386)x1 | 42 | 19+0 | Abnormal NIPT | - | TOP |
|  | arr[hg19]17p11.2(16,657,318-20,417,235)x1 | 35 | 16+0 | Increased NT | - | TOP |
|  | arr[hg19]22q11.21(18,648,855-21,800,471)x1 | 35 | 24+0 | Urogenital malformations | - | TOP |
|  | arr[hg19]Xq22.3q28(107,912,179-155,233,098)x1 | 27 | 21+0 | Abnormal NIPT | - | TOP |
|  | arr[hg19]4p16.3p16.1(68,345-8,530,222)x1 | 32 | 24+0 | Cardiovascular malformations | - | TOP |
|  | arr[hg19]6q25.1q27(152,176,966-170,914,297)x1；arr[hg19]11q24.3q25(130,308,334-134,937,416)x3 | 30 | 16+4 | Abnormal NIPT | - | TOP |
|  | arr[hg19]17p12(14,099,564-15,484,335)x1 pat | 43 | 22+3 | CNS malformations | - | TOP |
|  | arr[hg19]17p12(14,098,951-15,482,833)x1 pat | 43 | 22+3 | Cardiovascular malformations | - | TOP |
|  | arr[hg19]Yq11.223(24,094,881-25,830,996)x0 | 27 | 17+5 | Increased NT | - | TOP |
|  | arr[hg19]22q11.21q11.22(21,464,763-22,495,971)x1 | 23 | 22+3 | Facial malformations | - | TOP |
|  | arr[hg19]4p16.3p15.2(68,345-22,917,134)x1； arr[hg19]14q32.12q32.33(94,599,556-107,284,437)x3；arr[hg19]11q22.1q25(100,024,019-134,937,416)x3 | 26 | 23+0 | Multiple malformations | - | LB |
|  | arr[hg19] q36.3(155,734,263-159,119,707)x1； arr[hg19]7q35q36.3(146,977,011-155,726,269)x3 | 29 | 21+0 | CNS malformations | - | TOP |
|  | arr[hg19]22q11.21(18,648,855-21,461,017)x3 | 37 | 17+5 | Increased NT | - | TOP |
|  | arr[hg19]22q11.21(18,636,749-21,800,471)x1 | 27 | 23+3 | Cardiovascular malformations | - | TOP |
|  | arr[hg19]5p15.33p15.31(113,576-6,908,240)x1 | 27 | 16+4 | Increased NT | - | IUD |
|  | arr[hg19]22q11.21(18,919,477-21,800,471)x3 | 22 | 16+2 | Increased NT | - | TOP |
|  | arr[hg19]Xp11.4p11.3(39,311,915-43,908,908)x1 | 34 | 16+0 | Abnormal tribunal history | - | TOP |
|  | arr[hg19]5q21.3q23.1(107,915,007-120,847,610)x1 | 40 | 16+4 | Abnormal NIPT | - | TOP |
|  | arr[hg19]6q25.1(149,390,349-149,824,950)x1 | 31 | 21+4 | Cardiovascular malformations | - | TOP |
|  | arr[hg19] 17q11.2 (28,980,561-30,369,402)x1 | 41 | 16+1 | Positive second trimester DS screening result | - | TOP |
|  | arr[hg19]12p13.33p13.32(173,786-3,543,326) x1dn | 32 | 24+1 | Abnormal NIPT | - | TOP |
|  | arr[hg19]8p23.3p23.1(158,048-7,044,046) x1，arr[hg19]8p23.1p11.22(12,532,885-39,678,723) x3 | 34 | 27+6 | CNS malformations | - | TOP |
|  | arr[hg19] Xp22.33 or Yp11.32(313,342-1,234,634 or 263,342-1,184,634) x1 | 41 | 16+1 | Positive second trimester DS screening result | - | TOP |
|  | arr[hg19]17q11.2(28,980,561-30,395,625)x1 | 41 | 16+1 | Positive second trimester DS screening result | - | TOP |
|  | LOH 11 | 26 | 22+6 | FGR | - | LB |
|  | arr[hg19]22q11.21q11.23(21,804,596-23,654,007)x1 | 30 | 23+0 | Facial malformations | - | TOP |
|  | arr[hg19]22q11.21q11.23(21,800,470-24,271,666)x3 pat | 26 | 22+3 | Cardiovascular malformations | - | LB |
|  | arr[hg19] Xq28(154,111,453-154,625,570)x1mat | 37 | 16+6 | Increased NT | - | LB |
|  | arr[hg19]22q11.21(18,631,364-21,800,471)x1 | 27 | 21+0 | Urogenital malformations | - | TOP |
|  | arr[hg19]15q24.1q24.2(72,930,195-76,071,744)x1 | 31 | 23+0 | CNS abnormalities | - | TOP |
|  | arr[hg19]Xp21.1(31,728,920-31,923,772)x0 | 27 | 22+4 | Family history of genetic disease | - | LB |
|  | arr[hg19]15q11.2(22,770,421-23,282,798)x1mat | 29 | 21+0 | Increased NT | - | LB |
|  | arr[hg19]Xp22.31(6,683,449-7,857,543)x1mat | 33 | 16+0 | Abnormal tribunal history | - | LB |
|  | arr[hg19]1q21.1q21.2(145,895,746-147,401,773)x3 | 46 | 19+2 | Chest malformations | - | LB |
|  | arr[hg19]15q11.2q13.1(23,290,787-28,560,664)x1 | 38 | 18+4 | Abnormal NIPT | - | TOP |
|  | arr[hg19]22q11.21(18,631,364-21,800,471)x1 | 33 | 23+6 | Cardiovascular malformations | - | TOP |
|  | arr[hg19]4p15.32p14(17,254,449-40,420,055)x3 dn; arr[hg19]10q21.3(64,964,440-66,413,630)x1 dn | 41 | 19+6 | Abnormal NIPT | - | TOP |
|  | arr[hg19]15q11.2q13.1(22,770,421-28,526,905)x3 | 41 | 16+0 | Advanced maternal age | - | LB |
|  | arr[hg19]22q11.21(18,919,477-21,459,713)x3 | 37 | 18+0 | Abnormal tribunal history | - | LB |
|  | arr[hg19]4p16.3p16.1(796,111-8,721,580) x1 | 40 | 16+0 | Abnormal CMA result | - | TOP |
|  | arr[hg19]3q22.1q29(129,731,739-197,851,444)x3; arr[hg19]8p23.3p23.2(158,048-5,524,545)x1 | 41 | 16+0 | Family history of genetic disease | - | TOP |
|  | arr[hg19]10q22.3q23.2(81,458,768-89,142,165)x1 | 28 | 21+4 | Cardiovascular malformations | - | TOP |
|  | arr[hg19]7q11.23(72,669,480-74,154,209)x1 | 37 | 23+0 | Urogenital malformations | - | TOP |
| ***Uncertain Result*** | ***85*** |  |  |  |  |  |
| VUS  （78） | arr[hg19]1q21.1 (145,382,123-145,775,966)×1 | 35 | 19+0 | Abnormal tribunal history | - | LB |
|  | arr[hg19] ]2q33.2q33.3(204,115,208-205,482,006)x3 | 34 | 16+0 | Abnormal tribunal history | - | LB |
|  | arr[hg19]4p16.1(9,663,649 - 10,660,896)×3 | 36 | 18+0 | Advanced maternal age | - | LB |
|  | arr[hg19]21q21.1q21.2（23,827,807 - 26,312,474）×3 | 35 | 20+0 | Advanced maternal age | - | LB |
|  | arr[hg19]2p12(78,484,700 - 80,089,819)×4 | 37 | 16+4 | Increased NT | - | LB |
|  | arr[hg19]9q21.12q21.33 (72,265,36687,478,135)hmz | 29 | 16+0 | Increased NT | - | LB |
|  | arr[hg19]14q23.2q23.3(64,048,751-64,920,398）x3 | 31 | 22+5 | Urogenital malformations | - | TOP |
|  | arr[hg19]21q11.2 (15,016,486-15,708,754)x3 | 28 | 22+0 | Urogenital malformations | - | IUD |
|  | arr[hg19]10p15.3（263,576-566,764）x 3 | 32 | 23+4 | Urogenital malformations | - | TOP |
|  | arr[hg19]X q13.1（68,923,953-69,567,137）x 3 | 25 | 22+0 | Urogenital malformations | - | LB |
|  | arr[hg19]7 p11.2(56,986,440-57,906,704)x 3 | 35 | 16+4 | Abnormal tribunal history | - | LB |
|  | arr[hg19]21q11.2q22.3(15,016,486-48,093,361)x2-3 | 41 | 16+0 | Positive second trimester DS screening result | 47,XN,+21[20]/46,XN[80] | TOP |
|  | arr[hg19] 8q12.1(57,062,619-61,569,436)x1， arr[hg19] 18q22.3(69,394,951-70,400,007)x3 | 22 | 22+0 | Abnormal NIPT | - | LB |
|  | arr[hg19]4q22.1q22.2(92,313,646-93,829,603)x3 | 27 | 16+4 | Abnormal tribunal history | - | LB |
|  | 16,17-LOH | 19 | 23+4 | Abnormal tribunal history | - | LB |
|  | arr[hg19] 6q26(162,594,940-163,217,212)x3 | 39 | 17+3 | Positive second trimester DS screening result | - | LB |
|  | arr[hg19]2q23.3q32.2(150,606,334-191,660,849) hmz，arr[hg19]5q14.1q14.3(78,927,017-88,159,285) hmz，arr[hg19]8q22.3q24.22(102,954,846-135,176,668) hmz | 28 | 16+5 | Family history of genetic disease | - | LB |
|  | arr[hg19] Yp11.2(3,864,133-6,107,721)x0； arr[hg19] Yp11.2(6,172,722-8,302,071)x0 | 25 | 23+0 | Multiple malformations | - | TOP |
|  | arr[hg19]17p13.3(1,139,336-1,457,528)x3 | 30 | 22+3 | Multiple malformations | - | TOP |
|  | arr[hg19]Xp22.33 or Yp11.32(482,581-1,700,121 or 432,581-1,650,121)x3 | 35 | 22+4 | Cardiovascular malformations | - | LB |
|  | arr[hg19] 17p13.3(169,218-575,504)x3 | 37 | 22+4 | Cardiovascular malformations | - | LB |
|  | arr[hg19]Xq21.31q21.32(91,634,275-92,593,665)x3 | 26 | 19+1 | Family history of genetic disease | - | LB |
|  | arr[hg19]20p12.1(12,742,919-13,338,336)x3 | 24 | 21+3 | Abnormal tribunal history | - | LB |
|  | arr[hg19]9p23p13.1(13,107,600-38,771,831) hmz； arr[hg19]9q21.11q22.31(71,013,799-95,657,711) hmz；arr[hg19]9q33.3q34.3(129,478,472-141,011,581) hmz | 26 | 23+2 | Abnormal NIPT | - | LB |
|  | LOH | 39 | 17+6 | Family history of genetic disease | - | LB |
|  | arr[hg19] 22q11.21(18,919,477-21,800,471)x3 | 30 | 16+2 | Positive second trimester DS screening result | - | LB |
|  | arr[hg19] 16p13.11(15,481,747-16,278,133)x3； arr[hg19]16p12.3(16,858,431-18,242,713)x3 | 33 | 22+0 | Cardiovascular malformations | - | TOP |
|  | arr[hg19] 16p11.2(29,428,531-30,176,508)x1 | 24 | 22+0 | Abnormal NIPT | - | LB |
|  | arr[hg19]Xq21.31q21.32(91,634,275-92,593,665)x3 | 34 | 17+5 | Abnormal NIPT | - | LB |
|  | arr[hg19]15q25.2q25.3(84,910,815-85,790,661)x1 | 30 | 19+3 | Increased NT | - | LB |
|  | LOH | 22 | 19+4 | Family history of genetic disease | - | LB |
|  | arr[hg19] Xp22.33(385,561-1,536,915)x3 | 31 | 18+4 | Increased NT | - | LB |
|  | LOH | 26 | 19+4 | Increased NT | - | LB |
|  | arr[hg19]18p11.31p11.23(5,694,649-7,974,486)x3 mat | 19 | 22+0 | Fetal tumor | - | TOP |
|  | arr[hg19]16p13.11(14,929,070-16,278,133)x3 | 33 | 19+0 | Family history of genetic disease | - | LB |
|  | arr[hg19]2q12.2q13(107,040,324-113,111,856)x3 mat | 28 | 17+0 | Abnormal NIPT result | - | LB |
|  | arr[hg19]17p13.3(964,097-2,429,886)x3 | 34 | 24+2 | Urogenital malformations | - | TOP |
|  | arr[hg19]16p11.2(29,351,826-30,190,029)x1 dn | 38 | 24+0 | Abnormal NIPT result | - | Lost |
|  | arr[hg19]15q25.2q26.3(83,759,214-102,397,317) hmz | 25 | 22+0 | Abnormal NIPT result | - | LB |
|  | arr[hg19]11q12.3q13.3(62,836,183-68,592,901) hmz；arr[hg19] 19p13.3(260,911-6,327,194) hmz | 41 | 17+0 | Abnormal tribunal history | - | LB |
|  | arr[hg19]Xp11.4q28(41,860,797-155,233,098)x2-3 (30%) | 26 | 22+2 | Facial malformations | - | TOP |
|  | arr[hg19]Xp11.23q22.1(46,406,863-102,151,216)x2-3； arr[hg19]Xq26.2q28(133,091,945-155,233,098)x2-3 | 26 | 21+0 | Multiple malformations | - | TOP |
|  | arr[hg19] 12p13.33(1,953,989-2,315,926)x3 pat | 30 | 18+5 | Cardiovascular malformations | - | Lost |
|  | arr[hg19] 15q21.3(53,177,488-54,155,564)x3 | 41 | 17+5 | Advanced maternal age | - | LB |
|  | arr[hg19] 16p11.2(29,591,326-30,243,606)x3 pat | 28 | 23+0 | Facial malformations | - | LB |
|  | arr[hg19] 16p11.2(28,508,988-30,177,240)x3 | 39 | 19+2 | Advanced maternal age | - | LB |
|  | arr[hg19]8q11.1q11.21(46,919,156-51,932,566) hmz；arr[hg19]9p24.3p23(216,123-12,914,396) hmz | 35 | 20+0 | Abnormal NIPT | - | LB |
|  | arr[hg19]Xq21.1(79,889,845-80,187,478)x2 mat | 32 | 18+0 | Increased NT | - | LB |
|  | arr[hg19]11p15.4p15.1(8,313,816-20,748,051) hmz；arr[hg19]11p11.2p11.12(46,063,033-51,550,787) hmz | 43 | 18+0 | Advanced maternal age | - | LB |
|  | arr[hg19] 13q21.2(60,321,686-60,709,021)x1 pat | 36 | 24+0 | Cardiovascular malformations | - | LB |
|  | arr[hg19]13q14.3q21.2(52,840,783-61,301,173)x3 pat | 36 | 20+2 | Abnormal NIPT | - | LB |
|  | arr[hg19] 7q21.2(91,269,594-91,785,021)x1 mat | 30 | 24+3 | Cardiovascular malformations | - | TOP |
|  | arr[hg19]6p12.1(53,311,803-56,719,541)x3 mat | 28 | 24+0 | Abnormal NIPT | - | Lost |
|  | arr[hg19]6p22.3(17,867,202-18,765,914)x1 | 35 | 20+0 | Abnormal NIPT | - | TOP |
|  | arr[hg19]18q23(74,368,288-77,807,767)x3 | 29 | 17+0 | Abnormal NIPT | - | Lost |
|  | LOH | 32 | 22+5 | CNS malformations | - | LB |
|  | arr[hg19]1q21.1q21.2(146,106,723-147,399,684)x3 | 32 | 19+0 | CNS malformations | - | TOP |
|  | arr[hg19]4q12q13.1(58,193,591-62,730,657)x4 | 36 | 19+5 | Abnormal NIPT | - | LB |
|  | LOH 6q | 34 | 17+1 | Abnormal NIPT | - | LB |
|  | arr[hg19]7q36.2(153,747,352-153,939,208)x1 pat | 23 | 16+4 | Increased NT | - | LB |
|  | arr[hg19]1p12p11.2(120,527,494-120,663,485)x1 | 27 | 16+3 | Positive second trimester DS screening result | - | LB |
|  | LOH 16 | 39 | 18+6 | Abnormal NIPT | - | TOP |
|  | arr[hg19]12q13.11q14.1(48,703,202-59,992,201) hmz | 25 | 23+0 | Facial malformations | - | TOP |
|  | arr[hg19]12p12.1p11.1(23,096,513-34,761,150) hmz；arr[hg19]12q11q12(38,190,102-45,518,178) hmz | 32 | 21+4 | Increased NT | - | LB |
|  | LOH 20p | 29 | 17+3 | Positive second trimester DS screening result | - | LB |
|  | arr[hg19]8p23.1(11,656,356-11,818,255)x3 | 28 | 23+2 | CNS malformations | - | TOP |
|  | LOH | 21 | 16+0 | Cardiovascular malformations | - | TOP |
|  | arr[hg19]10q11.22(46,293,590-48,126,560)x1 | 21 | 16+2 | Increased NT | - | IUD |
|  | arr[hg19]4q12q13.1(58,193,591-62,730,657)x3 | 28 | 20+0 | Abnormal NIPT | - | LB |
|  | arr[hg19]2q36.3(226,485,047-228,045,315)x3 dn | 45 | 17+0 | Advanced maternal age | - | LB |
|  | arr[hg19]6p25.1p24.3(5,447,413-8,258,235)x1； arr[hg19]6q14.1q15(83,509,412-88,843,867)x1 | 35 | 16+3 | Advanced maternal age | - | LB |
|  | arr[hg19]16p13.11p12.3(15,325,072-18,157,612)x3 | 27 | 19+1 | Abnormal NIPT | - | LB |
|  | arr[hg19]6p25.3p24.2(203,877-11,133,062)x2 hmz | 33 | 21+5 | Abnormal NIPT | - | LB |
|  | arr[hg19] 2p12(75,447,327-76,712,612)x3 | 30 | 23+0 | Abnormal CMA result | - | LB |
|  | arr[hg19]10q21.1(56,907,768-57,951,612)x1; arr[hg19]10q21.1(58,128,333-59,340,551)x1 | 25 | 17+5 | Family history of genetic disease | - | LB |
|  | arr[hg19] 4q35.2(189,399,584-190,957,460)x1 pat | 30 | 17+5 | Abnormal karyotyping result | - | TOP |
|  | arr[hg19] 10q11.22(46,293,590-48,126,560)x1 | 28 | 17+1 | Positive second trimester DS screening result | - | LB |
| Likely Benign  (7) | LOH | 27 | 23+3 | Urogenital malformations | - | LB |
|  | arr[hg19]14q32.33(106,246,288-106,751,178)x3 pat | 27 | 19+0 | Abnormal tribunal history | Normal | LB |
|  | arr[hg19]2q12.2q13(107,006,550-111,370,025)x3 mat | 27 | 21+0 | Abnormal NIPT | - | LB |
|  | arr[hg19]15q25.3(86,026,393-87,263,409)x3 mat | 39 | 16+0 | Abnormal NIPT | - | Lost |
|  | arr[hg19]16p13.11(14,920,864-16,309,046)x3 mat | 43 | 18+2 | Advanced maternal age | - | LB |
|  | arr[hg19]Xp22.31(6,455,150-8,135,568)x2 | 27 | 17+0 | Increased NT | - | LB |
|  | arr[hg19]Xp22.31(6,449,836-8,135,568)x2 | 27 | 17+0 | Increased NT | - | LB |
| Total | 420 |  |  |  |  |  |

Abbreviations: CNV-Copy number variants; PTB-Preterm birth; IUD-Intrauterine death; FGR-Fetal growth restriction; NIPT-Noninvasive prenatal testing; CMA-Chromosomal microarray analysis; CNS malformations-Central nervous system malformations; VUS-Variants of uncertain significance; LB-Live birth; TOP-Termination of the pregnancy.
